# Complete functional mapping of infection- and vaccine-elicited antibodies against the fusion peptide of HIV

**DOI:** 10.1371/journal.ppat.1007159

**Published:** 2018-07-05

**Authors:** Adam S. Dingens, Priyamvada Acharya, Hugh K. Haddox, Reda Rawi, Kai Xu, Gwo-Yu Chuang, Hui Wei, Baoshan Zhang, John R. Mascola, Bridget Carragher, Clinton S. Potter, Julie Overbaugh, Peter D. Kwong, Jesse D. Bloom

**Affiliations:** 1 Basic Sciences Division and Computational Biology Program, Fred Hutchinson Cancer Research Center, Seattle, Washington, United States of America; 2 Molecular and Cellular Biology PhD program, University of Washington, Seattle, Washington, United States of America; 3 Division of Human Biology and Epidemiology Program, Seattle, Washington, United States of America; 4 Vaccine Research Center, National Institute of Allergy and Infectious Diseases, National Institutes of Health, Bethesda, Maryland, United States of America; 5 National Resource for Automated Molecular Microscopy, Simons Electron Microscopy Center, New York Structural Biology Center, New York, New York, United States of America; King’s College London, UNITED KINGDOM

## Abstract

Eliciting broadly neutralizing antibodies (bnAbs) targeting envelope (Env) is a major goal of HIV vaccine development, but cross-clade breadth from immunization has only sporadically been observed. Recently, Xu et al (2018) elicited cross-reactive neutralizing antibody responses in a variety of animal models using immunogens based on the epitope of bnAb VRC34.01. The VRC34.01 antibody, which was elicited by natural human infection, targets the N terminus of the Env fusion peptide, a critical component of the virus entry machinery. Here we precisely characterize the functional epitopes of VRC34.01 and two vaccine-elicited murine antibodies by mapping all single amino-acid mutations to the BG505 Env that affect viral neutralization. While escape from VRC34.01 occurred via mutations in both fusion peptide and distal interacting sites of the Env trimer, escape from the vaccine-elicited antibodies was mediated predominantly by mutations in the fusion peptide. Cryo-electron microscopy of four vaccine-elicited antibodies in complex with Env trimer revealed focused recognition of the fusion peptide and provided a structural basis for development of neutralization breadth. Together, these functional and structural data suggest that the breadth of vaccine-elicited antibodies targeting the fusion peptide can be enhanced by specific interactions with additional portions of Env. Thus, our complete maps of viral escape both delineate pathways of resistance to these fusion peptide-directed antibodies and provide a strategy to improve the breadth or potency of future vaccine-induced antibodies against Env’s fusion peptide.

## Author summary

A major goal of HIV-1 vaccine design is to elicit antibodies that neutralize diverse strains of HIV-1. Recently, some of us elicited such antibodies in animal models using immunogens based on the epitope of a broad antibody (VRC34.01) isolated from an infected individual. Further improving these vaccine-elicited antibody responses will require a detailed understanding of how the resulting antibodies target HIV’s envelope protein (Env). Here, we used mutational antigenic profiling to precisely map the epitopes of two vaccine-elicited antibodies and the template VRC34.01 antibody. We did this by quantifying the effect of all possible amino acid mutations to Env on antibody neutralization. Although all antibodies target a similar region of Env, we found clear differences in the functional interaction of Env with these vaccine- and infection-elicited antibodies. We combined these functional data with structural analyses to identify antibody–Env interactions that may contribute to the relatively greater breadth of the infection-elicited antibody and could improve the breadth of vaccine-elicited antibodies. These data thereby help to refine vaccination schemes to achieve broader responses.

## Introduction

The isolation of broadly neutralizing antibodies (bnAbs) capable of neutralizing diverse clades of HIV-1 has invigorated hopes of developing a broadly protective antibody-based HIV vaccine [1,2]. Epitope mapping of bnAbs has revealed a number of conserved sites of vulnerability on Env, and designing immunogens to elicit antibodies that target such sites is a promising vaccine strategy [3,4]. Structural characterization of the epitope of bnAb N123-VRC34.01 (subsequently referred to as VRC34.01) revealed that the N terminus of the fusion peptide (FP) is one such site of vulnerability [5]. A number of additional bnAbs that also partially target this epitope have been characterized [6–10], suggesting it may be a promising vaccine target.

Xu et al (2018) [11] used the VRC34.01 epitope as a template to design vaccine approaches that elicited antibodies to the fusion peptide capable of neutralizing diverse strains of HIV-1. The broadest of these vaccine-elicited fusion peptide-directed antibodies (vFP antibodies) derive from a class of murine antibodies, the vFP1 class, whose members share similar B cell ontogenies and structural modes of recognition. While the murine vFP1 class antibodies share VH gene sequence homology with the human VH gene of VRC34.01 (S1 File), the binding mode and antibody contact sites of the vFP antibodies are different from that of VRC34.01 [11]. Here we focus primarily on two of these antibodies, 2712-vFP16.02 and 2716-vFP20.01, which have breadths of 31% and 27% respectively, on a 208-strain cross-strain panel [11]. The vFP16.02 antibody was elicited by a BG505 Env trimer prime and three fusion peptide-carrier protein conjugate (FP-KLH) boosts in two-week intervals, and vFP20.01 was elicited via a similar scheme with an additional trimer boost. Antibodies were isolated via the generation of hybridomas three weeks after the last boost. The breadth of these antibodies rivals infection-elicited bnAbs such as 2G12 [12], although these vFP antibodies have lower affinity for Env than the original VRC34.01 bnAb [11]. To improve breadth and potency in further rounds of structure-based vaccine design, we undertook studies to define precisely the epitope specificities of the vFP antibodies, to delineate viral pathways of functional resistance, and to understand how these differ from the original template VRC34.01 bnAb.

## Results

We used mutational antigenic profiling [13] to quantify how all single amino-acid mutations at each residue of Env affected the neutralization of replication-competent HIV by VRC34.01 and the two vaccine-induced antibodies, vFP16.02 and vFP20.01. Specifically, for each antibody, we neutralized libraries [14] of viruses carrying all amino-acid mutations to the ectodomain and transmembrane domain of the BG505.T332N Env at an ~IC_95_ antibody concentration. For each of the possible 12,730 Env mutations (670 mutagenized sites × 19 amino acid mutations), we calculated the enrichment of the mutation in the antibody-selected condition relative to a non-selected mutant virus library, a quantity that we term *differential selection* [13] (S1A Fig). This entire process was performed in full biological replicate, beginning with independent generation of the proviral plasmid mutant libraries. The results from the two replicates were well correlated (S1B and S1C Fig). For the remainder of the paper, we present the averaged data from these two replicates.

**Fig 1:**
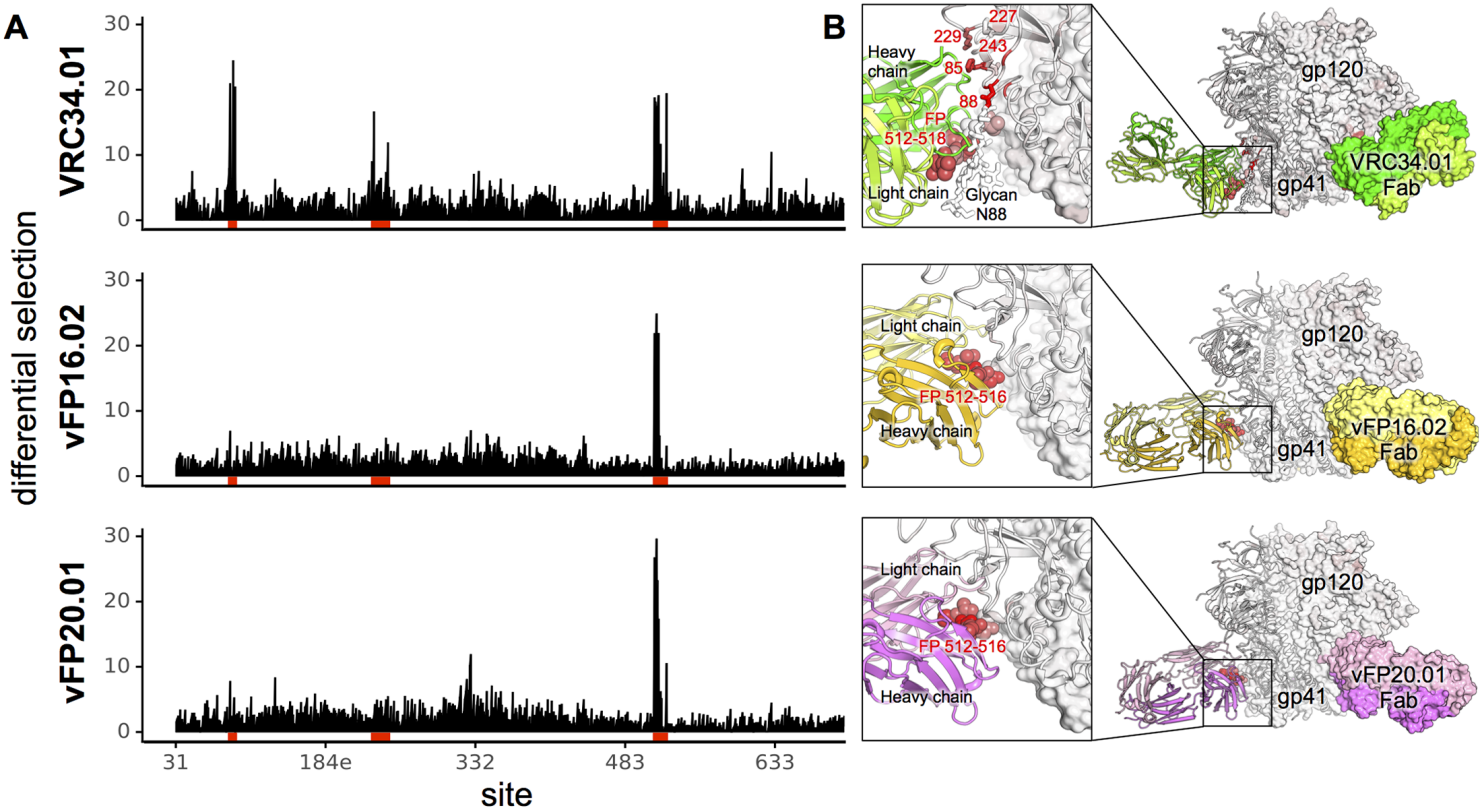
Complete escape profile of natural and vaccine elicited fusion peptide antibodies. **A**. The positive site differential selection is plotted across the length of the mutagenized portion of Env for each antibody. **B**. Structural representation of the FP-directed antibody epitope on BG505 Env trimer. The Env trimer is colored from white to red according to the positive differential selection at each site. Cartoon diagram of Fab-trimer complexes were adapted from crystal structure (VRC34.01) and cryoEM structure (vFP16.02 and vFP 20.01), with one Fab-protomer shown in ribbon and the other two Fab-protomers shown as surface. Labeled fusion peptide residues are shown in spheres. Detail of the Fab-trimer interaction was shown in the inset.

**Fig 2:**
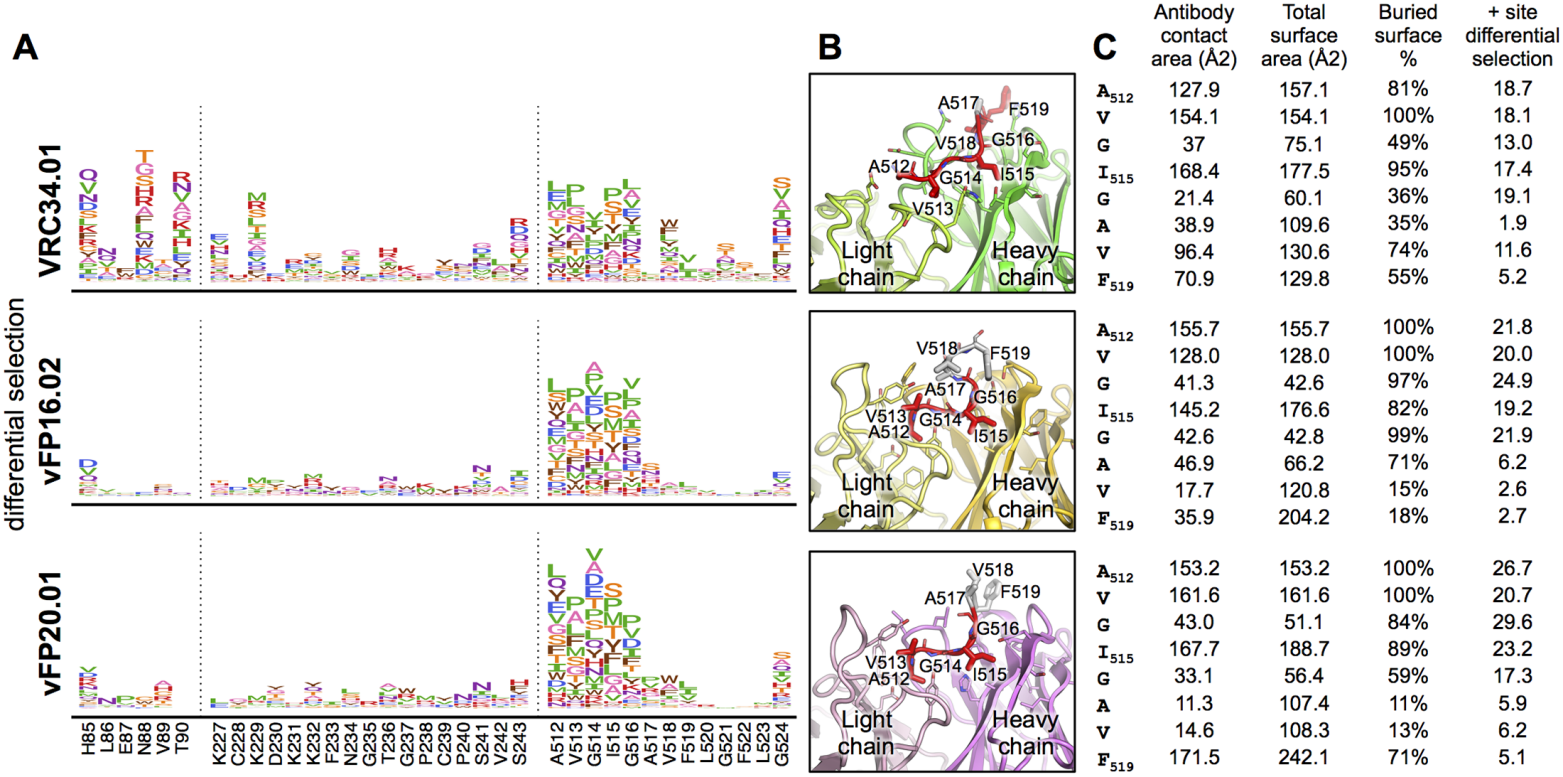
The mutation-level escape profiles agree with structural characterizations of the fusion peptide epitope. **A**. The mutation-level escape profile for regions with considerable differential selection by at least one antibody (regions with red underlay in Fig 1A). Left to right, these include the N88 glycosylation motif and neighboring area, surface exposed gp120 residues that interact with VRC34.01, and most of the fusion peptide. The height of each amino acid is proportional to the logarithm of the relative enrichment of that mutation in the antibody selected condition relative to the non-selected control. **B**. Fab-FP peptide crystal structures of each antibody. Antibody Fabs were colored as in Fig 1B, FP residues with significant differential selection to each antibody were colored in red. **C**. Total surface, antibody-contact surface (determined from co-crystal structures shown in Fig 2B), and positive site differential selection for each antibody.

It was immediately apparent that viruses with mutations in the N terminus of the fusion peptide (sites 512–516) robustly escaped all three antibodies (Figs 1A, S2–S4). However, there were also obvious differences between VRC34.01 and the two vaccine-elicited antibodies. While viral escape was concentrated in the fusion peptide for the vFP antibodies, there were a number of additional sites in Env where mutations allowed for escape from VRC34.01. Prior structural and functional characterization of VRC34.01 have revealed that it interacts with both the N terminus of the fusion peptide and the N-linked glycan at residue 88 [5]. The results of these prior structural and functional studies are highly correlated with our mutational antigenic profiling data (S2 and S5 Figs). Mutational antigenic profiling also revealed additional sites of viral escape from VRC34.01, including residues 84, 85, 227, 229, 241 and 243, which are all proximal to the fusion peptide (Fig 1B). We also observed a modest but reproducible signal for escape from vFP16.02 and vFP20.01 via introducing a glycosylation motif with S241N, where a glycan would cause steric hindrance to the approach of these antibodies, and via mutations at site 85 (Figs 2A, S3 and S4).

Since we measured the effect of every amino acid at every site, we could examine viral escape at a *mutation* rather than *site* level (Fig 2A). For example, V518W, V518M, and V518L resulted in drastic escape from VRC34.01 neutralization, while V518A had no effect (S2 and S5 Figs, and Kong et al (2016) [5]). Further, while the mutational antigenic profiling results agreed with alanine and glycine scans of the fusion peptide [11], the complete escape profiles identified additional escape mutants at nearly all of the fusion peptide residues that contact each antibody. This mutation-level mapping was especially informative for understanding the mechanistic basis for escape from neutralization. For example, hydrophobic amino acids were among the most enriched escape mutants at site 512–516 for all three antibodies. This may reflect their ability to eliminate antibody binding while still allowing for the fusion peptide to be efficiently inserted into the host cell plasma membrane during fusion.

Even within the fusion peptide region, there are differences between the template, infection-elicited bnAb and the vaccine-elicited antibodies. Escape from VRC34.01 could also occur via mutations at sites 518 and 521, whereas these mutations had little impact on the vaccine-elicited antibodies. These residue level differences in escape within the FP agree with structural interactions; within FP peptide-Fab co-crystal structures, the percent buried surface area of each residue tracks well with the extent of escape for each antibody (Fig 2B and 2C). Likewise, the fusion peptide dominated the epitope of antibodies vFP16.02 and vFP20.01 bound to Env, with the cryo-EM reconstructions showing strongest electron density of the bound antibody around the fusion peptide [11], with residues 512–519 accounting for ~63% and ~56% of the total protein epitope surface for vFP16.02 and vFP20.01, respectively (S1 Table). However, these data also highlighted how not all structural contacts contribute equally to binding. Cryo-EM (vFP16.02 and vFP20.01) and crystal (VRC34.01) structures of each Fab bound to HIV-1 Env showed contacts of the antibodies with residues 517, 519, and 520 of the fusion peptide of the trimeric Env (Fig 1B, S1B and S1C Table). Mutational antigenic profiling suggested that these residues were less critical to escape than residues 512–516.

We also found an unexpected site of viral escape at site 524 that was shared between all three antibodies. Although Gly 524 is distal to the epitope and does not directly contact any of the antibodies, it is part of a structural motif conserved in all three antibody-Env structures, involving a tight turn that allows Phe 522 to anchor the flexible fusion peptide via van der Waals interactions to α6 helix of gp41, and β5 and β7 strands of gp120 (S6 Fig) [5,11]. Interestingly, mutations at site 524 mediated escape from each antibody to differing extents (Fig 2A). Thus, these distal mutations likely altered the presentation or conformational dynamics of the N terminus of the fusion peptide, an observation relevant to immunogen design.

Sequence analysis of sensitive and resistant pseudoviruses provides an alternative approach to identifying naturally occurring individual Env mutations that were associated with resistance. Analysis of the FP sequences of a panel of 208 sensitive and resistant pseudoviruses identified mutations at sites 513 and 515 that were associated with resistance to the vFP antibodies, while mutations at sites 513, 514, 518, and 519 were associated with resistance to VRC34.01 (S7 Fig). More precisely, in the case of VRC34.01, the sequence-neutralization association analysis revealed that methionine and leucine at position 518 and isoleucine at position 519 were significantly associated with resistance, which were among the escape mutations identified by the antigenic profiling analysis (Fig 2A). However, the known critical glycosylation motifs such as N88 or N611 were not detected to be associated with antibody sensitivity in analysis of the pseudovirus panel. This is likely due to N88 and N611 being highly conserved in the panel of 208 pseudoviruses. While the resistance analysis was limited to mutations present at sufficient numbers in the pseudovirus panel, it nevertheless allowed for an examination of the effect of each mutation in diverse strains. These results agreed with the comprehensive escape profiles and highlighted naturally occurring diversity that may present a challenge to fusion peptide-targeting vaccines.

**Fig 3:**
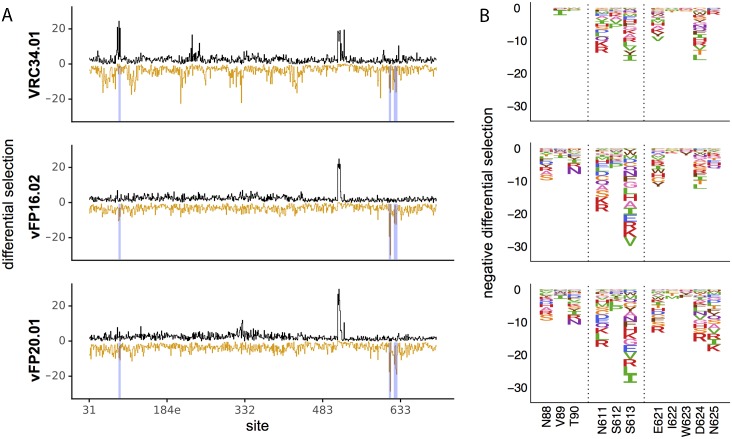
Mutations that better expose the fusion peptide are selected against during antibody treatment. **A**. The positive (black) and negative (orange) site differential selection is plotted across the length of the mutagenized portion of Env for each antibody. **B**. The negative differential selection for regions of interest (regions highlighted in blue in 1A). Left to right, these include the N88 glycosylation motif, the N611 glycosylation, and surface exposed gp41 sites that consistently have mutations depleted upon antibody selection. The height of each amino acid is proportional to the logarithm of the relative depletion of that mutation in the antibody selected condition relative to the non-selected control.

Mutational antigenic profiling also identified mutations distal to the epitope that were depleted, rather than enriched, during antibody selection relative to the non-selected libraries (Fig 3). These include mutations that eliminated the N611 glycosylation motif, indicating viruses that lack the N611 glycan are more sensitive to neutralization by these antibodies. To a lesser extent, we observed the same phenotype at the N88 glycosylation motif for the vFP antibodies but not for VRC34.01 (Fig 3A and 3B). These results are consistent with the fact that binding of VRC34.01 is dependent on the N88 glycan [5], and disrupting the N88 and N611 glycosylation motifs individually in BG505 pseudoviruses results in more potent neutralization by vFP16.02 and vFP20.01 [11]. Numerous mutations throughout Env were depleted during VRC34.01 selection, while far fewer mutations were depleted during vFP16.02 and vFP20.01 selection (Fig 3A). For all three antibodies, sites 621 and 624 were amongst those under the strongest negative differential selection (Fig 3A and 3B). These surface-exposed gp41 sites, which neighbor the FP epitope but do not directly interact with any of the antibodies, could allosterically affect the conformation and/or presentation of the fusion peptide or neighboring glycans. Indeed, site 621 is part of a surface salt bridge network that could be important for maintaining local structure (S8 Fig) [11]. While the mechanism of this phenomenon is yet to be fully elucidated, introducing these mutations into Env trimers may be a way to increase the exposure of the fusion peptide in trimer immunogens.

Our mutational antigenic profiling may also help to contextualize prior structural analyses [5,11]. vFP antibodies have a restricted angle of approach and substantial interactions with glycans at residues N88, N295, N448, and N611 [11], but these data show that eliminating these glycans did not result in viral escape. Thus, these antibodies likely approach the fusion peptide at an angle that accommodates or avoids steric clashes with glycans. We hypothesize that the vFP antibodies’ lack of direct reliance on glycans is a result of the vaccination regimen, where responses were induced in part with FP-KLH immunogens that lacked appropriately positioned glycans.

Further, vFP16.02 and vFP20.01’s focused recognition of the fusion peptide’s N terminus is also reflected by their Env-bound cryo-EM reconstructions [11]. Reduced electron density for the antibody outside of this region around the fusion peptide indicated greater conformational variability as a result of being less constrained by interactions with Env.

**Fig 4:**
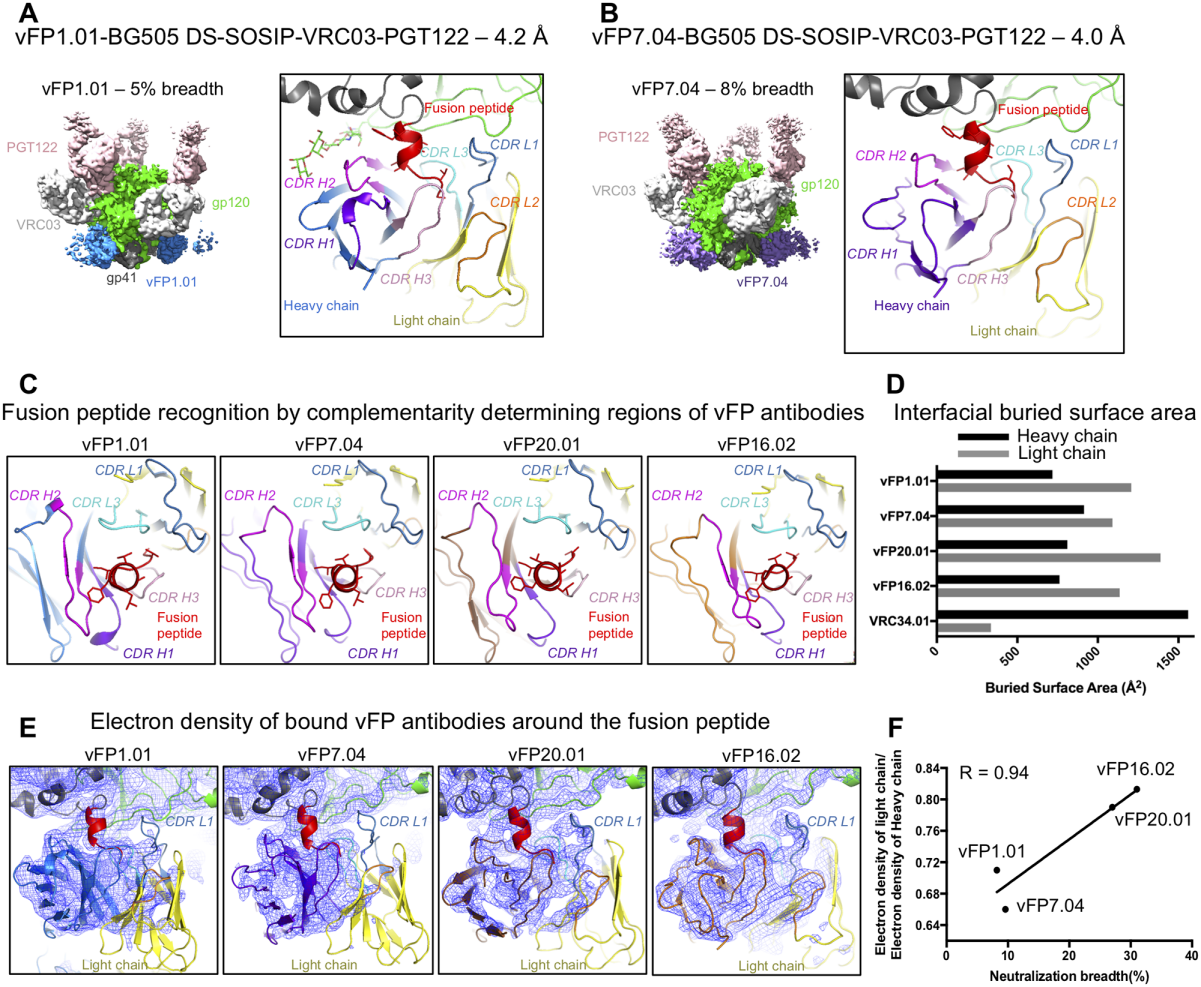
Structural basis for development of neutralization breadth of vFP antibodies. **A**. Left. CryoEM map of quaternary complex with antibody vFP1.01, segmented by components at a contour level that allowed visualization of Fv domains of antibodies. Right. Zoom-in of the fusion peptide region showing vFP1.01 bound with its CDR loops color-coded. **B**. Same as A, but with vFP7.04. **C**. Fusion peptide recognition by vFP1.01, vFP7.04, vFP16.02 and vFP20.01. **D**. Surface area buried at Env interface by the heavy and light chains of vFP1.01, vFP7.04, vFP20.01, vFP16.02 and VRC34. **E**. vFP antibody-bound fusion peptide with electron density from the cryo-EM reconstructions shown as blue mesh. All maps were low pass filtered to 4.5 Å. **F**. Light chain/heavy chain electron density ratio vs. neutralization breadth. The Pearson’s R correlation coefficient is shown.

To further understand the structural basis for development of antibody breadth for the vaccine-elicited antibodies, we determined cryo-EM structures of two additional vFP antibodies of the vFP1 class, vFP1.01 and vFP7.04 (8% and 10% breadth, respectively), in complex with BG505 DS-SOSIP Env trimer (Fig 4, S9 Fig, S1 and S2 Tables). We reasoned that an investigation of antibodies of the same class, but different breadth, might provide more sensitive identification of factors that increased breadth, which might be obscured if we compared antibodies from different classes. By using a similar strategy as was adopted for the cryo-EM reconstructions of Env-bound complexes of vFP16.02 and vFP20.01 [11], we obtained cryo-EM reconstructions, at overall resolutions of 4.2 Å and 4.0 Å, respectively, of vFP1.01-BG505 DS-SOSIP-VRC03-PGT122 and vFP7.04-BG505 DS-SOSIP-VRC03-PGT122 complexes, where the antigen binding fragments (Fabs) of antibodies PGT122 and VRC03 were added to increase the size of the complex and to provide fiducial markers allowing better particle visualization and alignment.

The cryo-EM reconstructions revealed vFP1.01 and vFP7.04 bound to a helical fusion peptide, in what appears to be a signature for this class of vaccine-elicited, murine antibodies (Fig 4, S9 and S10 Figs). By contrast, VRC34.01 binds an extended conformation of the fusion peptide. The ability of the fusion peptide to adopt different conformations demonstrates substantial structural plasticity with residue Phe 522 anchoring a highly flexible fusion peptide N terminus to the rest of the Env. In all four vaccine-elicited antibodies, vFP1.01, vFP7.04, vFP16.02 and vFP20.01, a conserved mode of recognition was observed, with both the heavy and light chains sequestering the fusion peptide N terminus and the light chain making additional contacts with gp120. Buried surface area calculations using the coordinates fitted and refined into the cryo-EM reconstructions revealed a 1.2–1.7-fold larger interfacial buried surface for the light chain than the heavy chain (Fig 4D). VRC34.01, on the other hand, showed a distinctly different mode of recognition with the heavy chain dominating recognition of the gp41 fusion peptide and gp120, burying ~4.5 fold more interfacial surface area than the light chain (Fig 4D), with the light chain interactions restricted to the N terminal tip of the fusion peptide and glycan 88 that forms a well-ordered interface involving both the heavy and light chains (S1A Table).

Although the light chain made a larger footprint on Env than the heavy chain in the vFP1.01, vFP7.04, vFP16.02 and vFP20.01 complexes, closer examination of the electron density revealed greater disorder in the light chain density in all the structures. This suggests local variability in the position of the bound light chain that was less constrained by its interactions with Env, whereas the heavy chain made a more stable interface, albeit with a smaller footprint, interacting primarily with the fusion peptide with some interactions with glycan 88. The most stable interface made by the light chain in all four complexes was via the CDR L3 loop interacting with the β0 region of gp120 and contacting gp120 residue His 85 (S11 Fig). We observed greater order in the light chains of vFP16.02 and vFP20.01 than of vFP1.01 and vFP7.04, especially around the CDR L1 loop. The relative extent of light chain order correlated with the breadth of these antibodies (Fig 4F), suggesting that for these vFP1-class antibodies a more stable light chain interface with Env could lead to increased neutralization breadth.

## Discussion

Overall, these in-depth delineations of the functional epitopes revealed similarities and differences between how VRC34.01 and the vaccine-elicited antibodies recognize Env. Escape from both vFP16.02 and vFP20.01 was mediated predominantly via mutations in the fusion peptide, whereas VRC34.01 neutralization was also affected by mutations at numerous additional sites.

The focused functional interface of the vaccine-elicited antibodies takes on added significance in the context of a unique structural observation: Env-bound vFP16.02 and vFP20.01 have reduced electron density outside of the paratope contacting the fusion peptide’s N terminus, indicating greater conformational heterogeneity. To further investigate this phenomenon, we determined two more vFP1-class antibody cryo-EM structures. These antibodies also displayed internal disorder, and the extent of relative light chain order correlated with breadth (Fig 4F). Increased light chain disorder may indicate weaker interactions with Env gp120. Since our mutational antigenic profiling clearly shows that VRC34.01 but not vFP16.02 or vFP20.01 has functional interactions outside the FP, we hypothesize that additional interactions with other regions of Env may increase the breadth of vFP antibodies. Consistent with this idea, the breadth of vFP antibodies is correlated with their affinity to Env trimer [11]. Our functional mapping does show modest but reproducible selection for escape mutations from vFP16.02 and vFP20.01 at site 85 in gp120 (Fig 2A), which stacks against their CDR L3 loops (S11 Fig). Thus, it does seem possible for the light chain of vaccine-elicited antibodies to derive advantageous interactions from this region of gp120.

Therefore, mapping *all* mutations that affect neutralization by infection and vaccine-elicited antibodies may help identify potentially desirable interactions to target with trimer boosts or rationale immunogen design. However, such additional interactions outside of the fusion peptide could increase affinity at the cost of reduced breadth if the additional contacts occur at Env sites that are variable in nature. Fortunately, most of the VRC34.01 escape mutations that that are outside of the fusion peptide are at sites largely conserved in nature (S12A and S12B Fig), suggesting that this tradeoff can be minimized. One strategy to elicit FP antibodies with additional Env interactions is to include additional Env trimer boosts. This could include Δ611 and/or Δ88 glycan trimers, which appear to increase the exposure of this epitope to vFP16.02 and vFP20.01, followed by wildtype trimers in hopes of guiding antibody lineages towards interacting with or avoiding clashes with these glycans. Alternatively, Env trimer immunogens that incorporate other mutations that appear to better expose this epitope (Fig 3) could be developed.

An alternative or parallel approach to increasing the breadth of fusion-peptide vaccine responses is to tailor the focused recognition of the fusion peptide to accommodate greater sequence diversity within this region [11]. This goal might be accomplished by immunizing with peptide immunogen pools that incorporate naturally found diversity within these sites [11]. Our data suggest that a peptide immunogen cocktail designed to elicit antibodies similar to vFP16.02 and vFP20.01 would need only to cover diversity at sites 512–516. Further, rather than designing cocktails that cover *all naturally occurring diversity* at sites 512–516, our data allows for the design of peptide cocktails that cover only those naturally occurring amino acid polymorphisms *that mediate escape from vFP antibodies*.

Within the fusion peptide, hydrophobic amino acids are among the most enriched escape mutations for all three antibodies (Fig 2A). This makes sense, since viral fusion requires insertion of Env’s hydrophobic fusion peptide into the target cell membrane. However, the numerous escape mutations mapped in our experiments contrast with the high sequence conservation of the fusion peptide’s N terminus in nature (S12B Fig). The much higher conservation of the fusion peptide in nature may be because there is relatively little natural pressure for mutations at these sites or might indicate the presence of additional constraints in nature that are not present in our cell-culture experiments. Prior work has shown that the BG505 Env used in this study tolerates many mutations at conserved fusion peptide sites under selection for viral replication in cell culture (S12C Fig) [14]. However, this mutational tolerance varies among strains, since Env from strain BF520 (another subtype A transmitted/founder Env) is much less tolerant of mutations at fusion peptide sites such as 512 and 516 (S12B–S12D Fig) [14].

Translating the promising potential of the these vFP antibodies into consistent, broadly protective responses will require iterative rounds of vaccine design and detailed evaluation of elicited responses. This study is an early step in this process and is limited to examining the neutralization sensitivities and structures of a small number of antibodies. Here, we do not evaluate numerous other antibody-mediated immune functions. Further, it is yet to be determined if additional Env interactions outside of the FP, similar to VRC34.01, will indeed result in greater breadth, or if increased affinity alone may drive breadth.

In conclusion, our mutational antigenic profiling of vFP antibodies and the template VRC34.01 bnAb, combined with analyses of additional vFP antibody structures, have provided insights that can help refine vaccination regimens. We precisely mapped previously unappreciated interactions between Env and the template bnAb VRC34.01, and our functional and structural analyses suggest that these interactions may play a role in VRC34.01’s relatively greater breadth and could possibly help improve the breadth or potency of anti-FP antibodies. Therefore, these complete maps of viral escape provide a detailed atlas to guide subsequent rounds of structure-based vaccine design.

## Materials and methods

### Antibody production

Mammalian codon-optimized genes encoding either antibody heavy chain or light chain were synthesized at Gene Universal Inc (Newark, DE). Antibody genes were cloned into mammalian expression vector pVRC8400. Antibodies were produced as described in Xu et al (2018) [11].

### Generation of mutant virus libraries

The generation and characterization of the BG505 mutant proviral DNA libraries, and the resulting functional mutant virus libraries have been described in detail previously [14,15]. Briefly, proviral DNA libraries that contained randomized codon-level mutations at sites 31–702 (HXB2 numbering) of BG505.W6M.C2.T332N *env* were independently generated. These, and accompanying wildtype controls, were independently transfected into 293T cells (ATCC). The transfection supernatant was passaged at a low MOI (0.01 TZM-bl infectious units/cell) in SupT1.CCR5 cells (James Hoxie), and the resulting genotype-phenotype linked mutant virus library was concentrated via ultracentrifugation. Env residues are numbered according to HXB2 reference numbering throughout.

### Mutational antigenic profiling

We have previously described Env mutational antigenic profiling using BF520 Env libraries [13]; a similar approach was taken here. Briefly, 5×10^5^ infectious units of two independent mutant virus libraries were neutralized with VRC34.01, vFP16.02, or vFP20.01 at an ~IC_95_ concentration (33 ug/mL, 500 ug/mL, or 500ug/mL of antibody, respectively) for one hour. Neutralized libraries were then infected into 1×10^6^ SupT1.CCR5 cells in R10 (RPMI [GE Healthcare Life Sciences; SH30255.01], supplemented with 10% FBS, 1% 200 mM L-glutamine, and 1% of a solution of 10,000 units/mL penicillin and 10,000 μg/mL streptomycin), in the presence of 100ug/mL DEAE-dextran. Three hours post infection, cells were spun down and resuspended in 1 mL fresh R10 without DEAE-dextran. At 12 hours post infection, cells were washed with PBS and non-integrated viral cDNA was isolated via miniprep. In parallel to each antibody selection, each mutant virus library was also subjected to a mock selection, and four 10-fold serial dilutions of each mutant virus library were also used to infect 1×10^6^ cells to serve as an infectivity standard curve. Selected and mock-selected viral cDNA was then sequenced with a barcoded subamplicon sequencing approach as previously described [14]. This approach involves introducing unique molecular identifiers during the Illumina library prep in order to further reduce the sequencing error rate.

The relative amount of viral genome that successfully entered cells was measured using *pol* qPCR[16]. A qPCR standard curve was generated based on each mutant virus library’s infectivity standard curve, from which the % neutralization for each antibody selected sample was calculated.

### Analysis of deep sequencing data

We used dms_tools2 version 2.2.4 (https://jbloomlab.github.io/dms_tools2/) to analyze the deep sequencing data and calculate the differential selection [17]. The differential selection statistic has been described in detail [13,18] and is further documented at https://jbloomlab.github.io/dms_tools2/diffsel.html. Sequencing of wildtype proviral DNA plasmid was used as the error control during the calculation of differential selection. Differential selection was visualized on logoplots rendered by dms_tools2 using weblogo [19] and ggseqlogo [20].

### Cryo-EM

HIV-1 Env BG505 DS-SOSIP and antibody Fab fragments were prepared as described previously [21]. For preparing the vFP1.01-BG505 DS-SOSIP-VRC03-PGT122 and the vFP7.04-BG505 DS-SOSIP-VRC03-PGT122 complexes, 4-5-fold molar excess of antibody Fab fragments of vFP1.01 and vFP7.04, respectively, as well as of VRC03 and PGT122, were incubated with HIV-1 Env BG505 DS-SOSIP at a final concentration of 0.3 mg/mL. To prevent aggregation during vitrification, 0.085 mM dodecyl-maltoside (DDM) was added to the sample, followed by vitrification using a semi-automated Spotiton V1.0 robot [22,23]. The grids used were specially designed Nanowire self-blotting grids with a Carbon Lacey supporting substrate [24]. Sample was dispensed onto these nanowire grids using a picoliter piezo dispensing head. A total of ~5 nL sample was dispensed in a stripe across each grid, followed by a pause of a few milliseconds, before the grid was plunged into liquid ethane.

Data was acquired using the Leginon system [25] installed on a Titan Krios electron microscope operating at 300kV and fitted with Gatan K2 Summit direct detection device. The dose was fractionated over 50 raw frames and collected over a 10-s exposure time. Individual frames were aligned and dose-weighted. CTF was estimated using the GCTF package [26]. Particles were picked using DoG Picker [27] within the Appion pipeline [28]. Particles were extracted from the micrographs using RELION [29]. 2D classification, ab initio reconstruction, heterogeneous refinement, and final map refinement were performed using cryoSparc [30]. Postprocessing was performed within RELION.

Fits of the Env trimer and Fab co-ordinates to the cryo-EM reconstructed maps were performed using UCSF Chimera [31]. The coordinates were refined by an iterative process of manual fitting using Coot [32] and real space refinement within Phenix [33]). Molprobity [34] and EMRinger [35] were used to check geometry and evaluate structures at each iteration step. Figures were generated in UCSF Chimera and Pymol (www.pymol.org). Electron density quantification and map-fitting cross correlations were calculated within UCSF Chimera. For electron density calculations maps were postprocessed using the larger mask used for refinement in cryoSparc. This mask encompassed the entire complex, including the more disordered antibody constant domains. The maps were then low pass filtered to 4.5 Å in EMAN2 before performing the electron density quantification calculations using the “Values at atom positions” function in Chimera. The electron density at the atom positions for the fitted models were then summed over the heavy and light chains (variable domains only). Map-to-model FSC curves were generated using EMAN2.

### Resistance analysis of 208-pseudovirus panel

We calculated the associations between the sequence variability of FP sequences at sites 512–519 and data from a 208-pseudovirus neutralization panel [36] using an in-house version of the approach implemented in R package SeqFeatR [37]. The resulting P-values were corrected using multiple testing method Holm.

### Data availability and source code

Open-source software that recapitulates the mutational antigenic profiling analysis, starting with the deep sequencing data through the calculation of selection metrics, is available at https://jbloomlab.github.io/dms_tools2/. The entire computational analysis, starting with downloading the deep sequencing reads through generating figures, is available as an executable IPython Notebook (S2 File) and at https://github.com/jbloomlab/MAP_Vaccine_FP_Abs. Differential selection values for each antibody are provided as S3–S5 Files. Illumina deep sequencing reads were deposited into the NCBI SRA as SRR6429862-SRR6429875.

The cryo-EM maps and fitted coordinates for vFP1.01-BG505 DS-SOSIP-VRC03-PGT122 and vFP7.04-BG505 DS-SOSIP-VRC03-PGT122 have been deposited to the RCSB database with accession numbers EMD-7622/PDB 6CUF and EMD-7621/PDB 6CUE, respectively.

## Supporting information

S1 Fig The mutational antigenic profiling pipeline. **A**. Schematic showing the mutational antigenic profiling pipeline. Genotype-phenotype linked mutant virus library, which has undergone functional selection, is neutralized by each antibody before infecting SupT1.CCR5 cells. The mutant virus library is also infected into cells in the absence of antibody selection. Viral cDNA is isolated from each condition and deep sequenced. Antibody escape mutants are enriched in the antibody selected condition relative to the non-selected control. **B**. The entire mutational antigenic profiling pipeline, including generating independent mutant proviral DNA libraries, was performed in duplicate. **C**. The positive site differential selection between biological replicates was well correlated for each antibody. The axes are labeled with the % remaining infectivity relative to the mock selected library for that replicate, measured via qPCR.
(TIFF)

S2 Fig The escape profile for VRC34.01. The height of each amino acid is proportional to the logarithm of the relative enrichment of that mutation in the antibody selected condition relative to the non-selected control. The wildtype BG505.T332N sequence is shown. Underlaid is the original functional mapping data from Kong et al (2016), where a number of mutant BG505 pseudoviruses (lacking the T332N mutation, unlike the BG505 Env used to generate to the mutant libraries) were tested in TZM-bl neutralization assays [5]. If a mutation was tested at a site, then a box is underlaid and colored according to the fold-change in IC_50_ relative to wildtype.
(TIFF)

S3 Fig The escape profile for vFP16.02. As described for S2 Fig, but for vFP16.02.
(TIFF)

S4 Fig The escape profile for vFP20.01. As described for S2 Fig, but for vFP20.01.
(TIFF)

S5 Fig Validation of mutational antigenic profiling. **A**. Correlation between the positive differential selection at a site and the log fold change in IC_50_ from a BG505 pseudovirus bearing a point mutant at that site (TZM-bl data from Kong et al (2016) [5], as described in S2 Fig). Note that the TZM-bl data is right censored, and it is possible for a mutation to affect the maximum percent neutralization rather than the IC_50_.
(TIFF)

S6 Fig F522 anchors the fusion peptide. A. Env from VRC34-bound complex with gp41 colored cyan, gp120 colored green and the fusion peptide colored red. Residues 522–524 of gp120 are shown in sticks. B. Same as a, but for Env from vFP16.02-bound complex and gp41 shown in dark gray. C. Overlay of VRC34-bound complex and vFP16.02-bound complex.
(TIFF)

S7 Fig FP sequence association with neutralization for vFP16.02, vFP20.01, and VRC34.01, based on a panel of 208 Env pseudoviruses. Bar plot shows the P-values of the association (calculated by Fisher’s exact test) between amino acid variation and neutralization at residues 512–519. Black bars highlight amino acid and site combinations that significantly associate with resistance to antibodies, while light-gray bars highlight sensitive ones. Amino acids valine and isoleucine at sites 513 and 515, respectively, are significantly associated with sensitivity, while isoleucine at position 513 and leucine at site 515 are significantly associated with resistance to vFP16.02. In the case of vFP20.01, residues valine, isoleucine and valine at sites 513, 515 and 518, respectively, are associated to sensitivity to vFP20.01, with only leucine at position 515 being significantly associated to resistance. For VRC34, valine at positions 513 and 518 as well as phenylalanine at site 519 are significantly associated to sensitivity to VRC34, while leucine and methionine at site 518, and isoleucine at site 519 are associated with resistance. A dotted red line corresponds to the adjusted P-value threshold of 0.05.
(TIFF)

S8 Fig A surface salt bridge network in gp41. Zoom-in of gp41 region showing a surface salt bridge network formed by residues E621, R617 and E634.
(TIFF)

S9 Fig Cryo-EM structural details for vFP1.01-DS-SOSIP-VRC03-PGT122 and vFP7.04-BG505 DS-SOSIP-VRC03-PGT122 complexes. **A**. Representative micrograph (left) with particles picked with DogPicker shown in red circles (right). **B**. Representative 2D class averages. **C**. Ab initio models generated using cryoSparc. **D**. Refined map in blue (for vFP1.01 complex) and purple (for vFP7.04 complex) with the mask used in RELION postprocessing shown in green. FSC plots and resolution values reported by RELION according to the gold standard FSC_0.143_ criterion (FSC_0.143_ shown as dotted line_._ **E**. Local resolution estimation. **F**. For density quantifications we used maps that were postprocessed using expanded solvent masks that encompassed the entire complex, including the more disordered constant domains. The FSC plots and overall resolutions of these maps are shown. The maps were then low-pass filtered to 4.5 Å for the density quantifications.
(TIFF)

S10 Fig Representative electron density snapshots for the vFP1.01-DS-SOSIP-VRC03-PGT122 and vFP7.04-BG505 DS-SOSIP-VRC03-PGT122 complexes. Electron density is shown as a blue mesh and the fitted model is shown as a cartoon with residue side-chains shown as sticks.
(TIFF)

S11 Fig Interaction of His 85 with CDR L3 loop of vFP Mabs. (TIFF)

S12 Fig Sequence conservation in nature and mutational tolerance in cell culture of the VRC34.01 functional epitope. **A**. The VRC34.01 escape profile is shown as in Fig 2A. **B**. The amino-acid frequencies in nature, calculated from the group M LANL Web Alignment. **C**. The BG505 amino-acid preferences under selection for viral replication in cell culture, as measured in Haddox et al 2018 [14]. Briefly, the height of each amino acid corresponds to how well tolerated that amino acid is for viral replication in cell culture. **D**. The BF520 amino-acid preferences under selection for viral replication in cell culture, as measured in Haddox et al 2018 [14]. For BF520, wildtype amino acids that differ from BG505 are colored red.
(TIFF)

S1 Table Buried surface area calculations of VRC34.01 and vFP antibodies bound to BG505 Env trimer. (TIFF)

S2 Table Cryo-EM data collection, refinement and validation statistics. (DOC)

S1 File Heavy and light chain sequences of antibodies studied. (FASTA)

S2 File Mutational Antigenic Profiling IPython notebook analysis. This zipped file contains the entire mutational antigenic profiling analysis. There is an html document of the completed analysis, an executable IPython notebook, and all of the necessary input data to execute the analysis.
(GZ) Click here for additional data file.

S3 File VRC34.01 differential selection. The VRC34.01 differential selection values for all measured mutations. The format is described at https://jbloomlab.github.io/dms_tools2/diffsel.html and in Dingens et al 2017 [13].
(CSV)

S4 File vFP16.02 differential selection. The vFP16.02 differential selection values for all measured mutations.
(CSV)

S5 File vFP20.01 differential selection. The vFP20.01 differential selection values for all measured mutations.
(CSV)
