# Supplementary figures and images for: Complete functional mapping of infection- and vaccine-elicited antibodies against the fusion peptide of HIV
Source: PLoS Pathog. 2018 Jul 5;14(7):e1007159. doi: 10.1371/journal.ppat.1007159 (PMC6049957; doi:10.1371/journal.ppat.1007159)

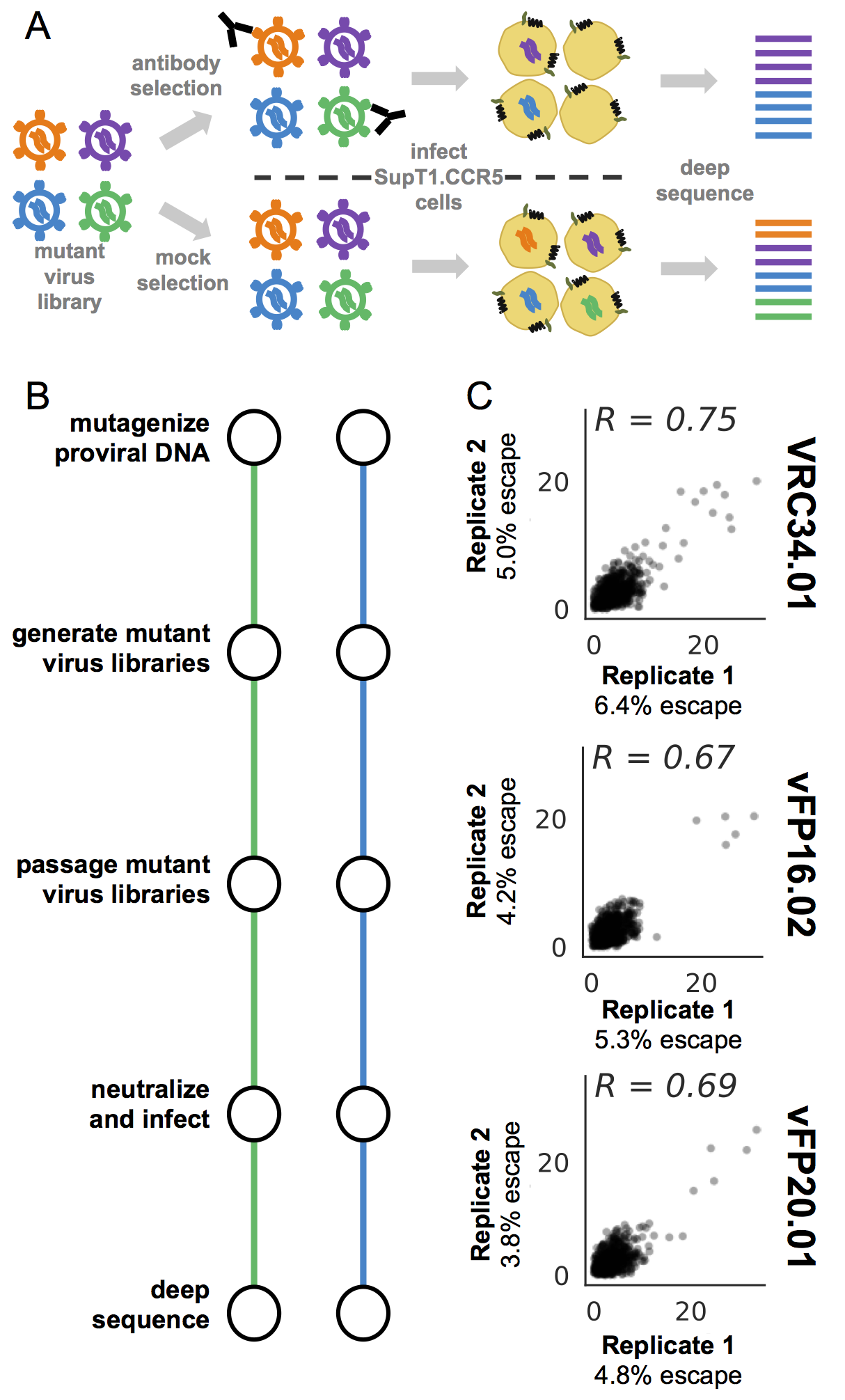

Supplement: S1 Fig — A. Schematic showing the mutational antigenic profiling pipeline. Genotype-phenotype linked mutant virus library, which has undergone functional selection, is neutralized by each antibody before infecting SupT1.CCR5 cells. The mutant virus library is also infected into cells in the absence of antibody selection. Viral cDNA is isolated from each condition and deep sequenced. Antibody escape mutants are enriched in the antibody selected condition relative to the non-selected control. B. The entire mutational antigenic profiling pipeline, including generating independent mutant proviral DNA libraries, was performed in duplicate. C. The positive site differential selection between biological replicates was well correlated for each antibody. The axes are labeled with the % remaining infectivity relative to the mock selected library for that replicate, measured via qPCR. (TIFF) [file ppat.1007159.s001.tiff]

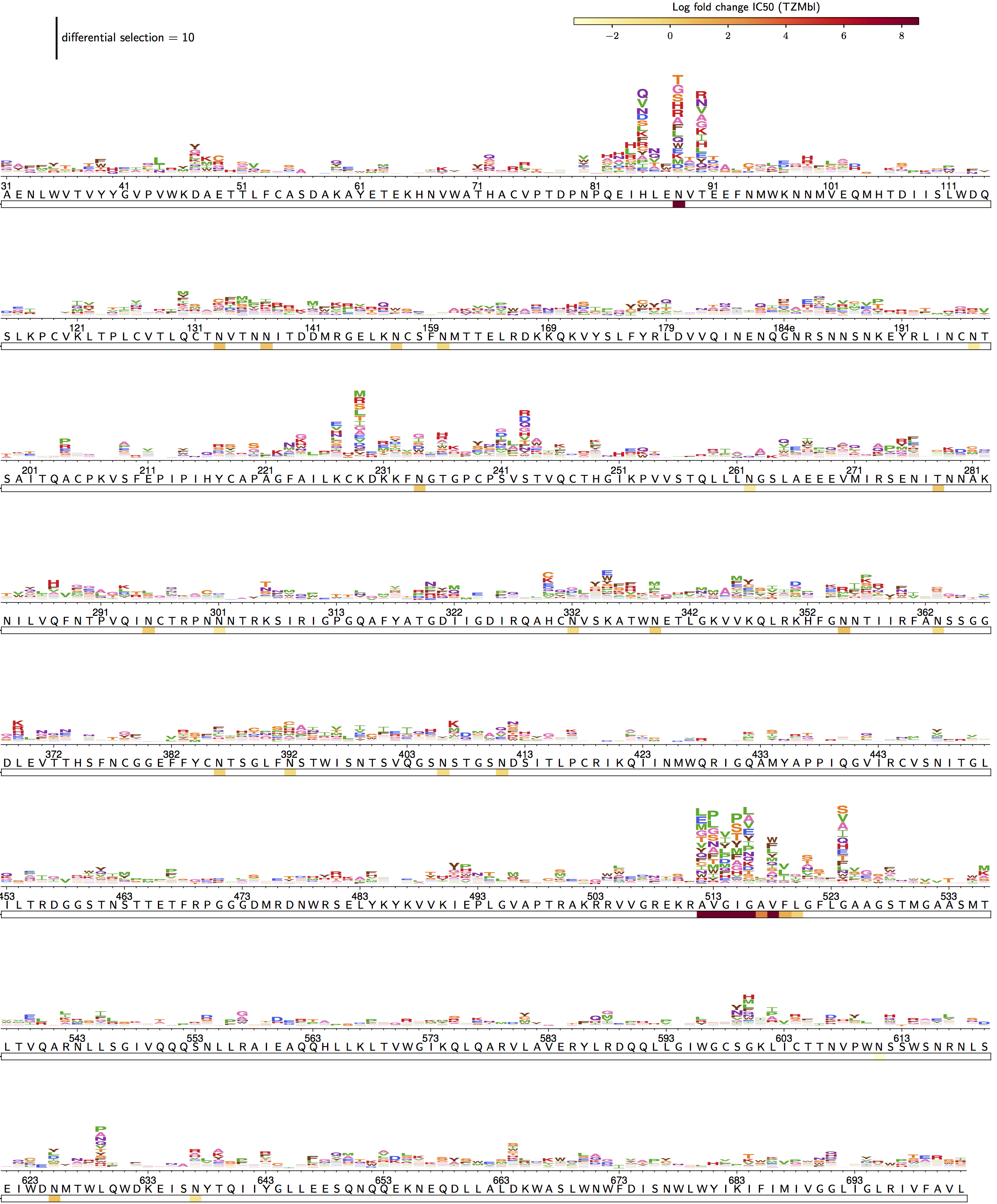

Supplement: S2 Fig — The height of each amino acid is proportional to the logarithm of the relative enrichment of that mutation in the antibody selected condition relative to the non-selected control. The wildtype BG505.T332N sequence is shown. Underlaid is the original functional mapping data from Kong et al (2016), where a number of mutant BG505 pseudoviruses (lacking the T332N mutation, unlike the BG505 Env used to generate to the mutant libraries) were tested in TZM-bl neutralization assays [5]. If a mutation was tested at a site, then a box is underlaid and colored according to the fold-change in IC50 relative to wildtype. (TIFF) [file ppat.1007159.s002.tiff]

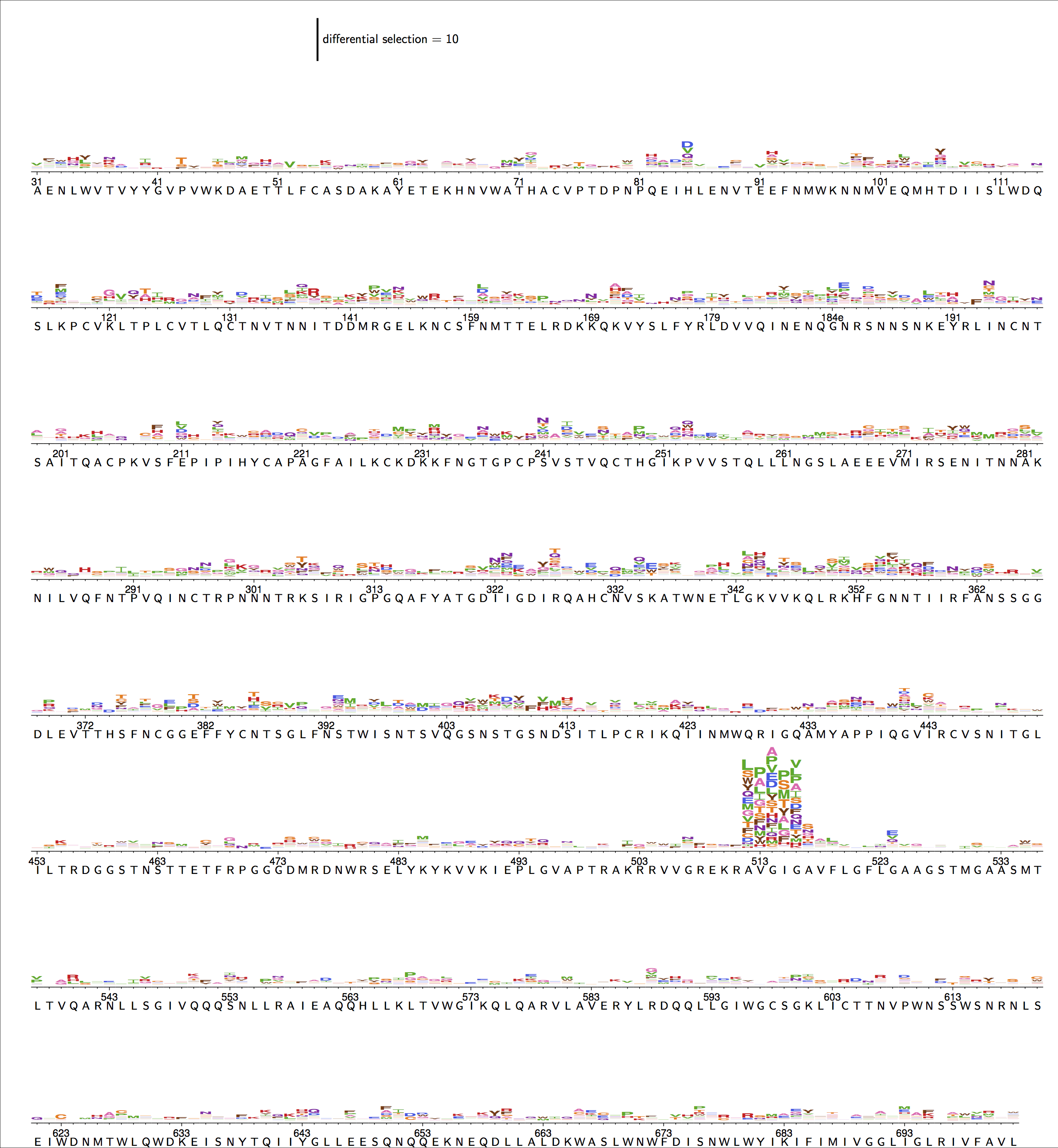

Supplement: S3 Fig — As described for S2 Fig, but for vFP16.02. (TIFF) [file ppat.1007159.s003.tiff]

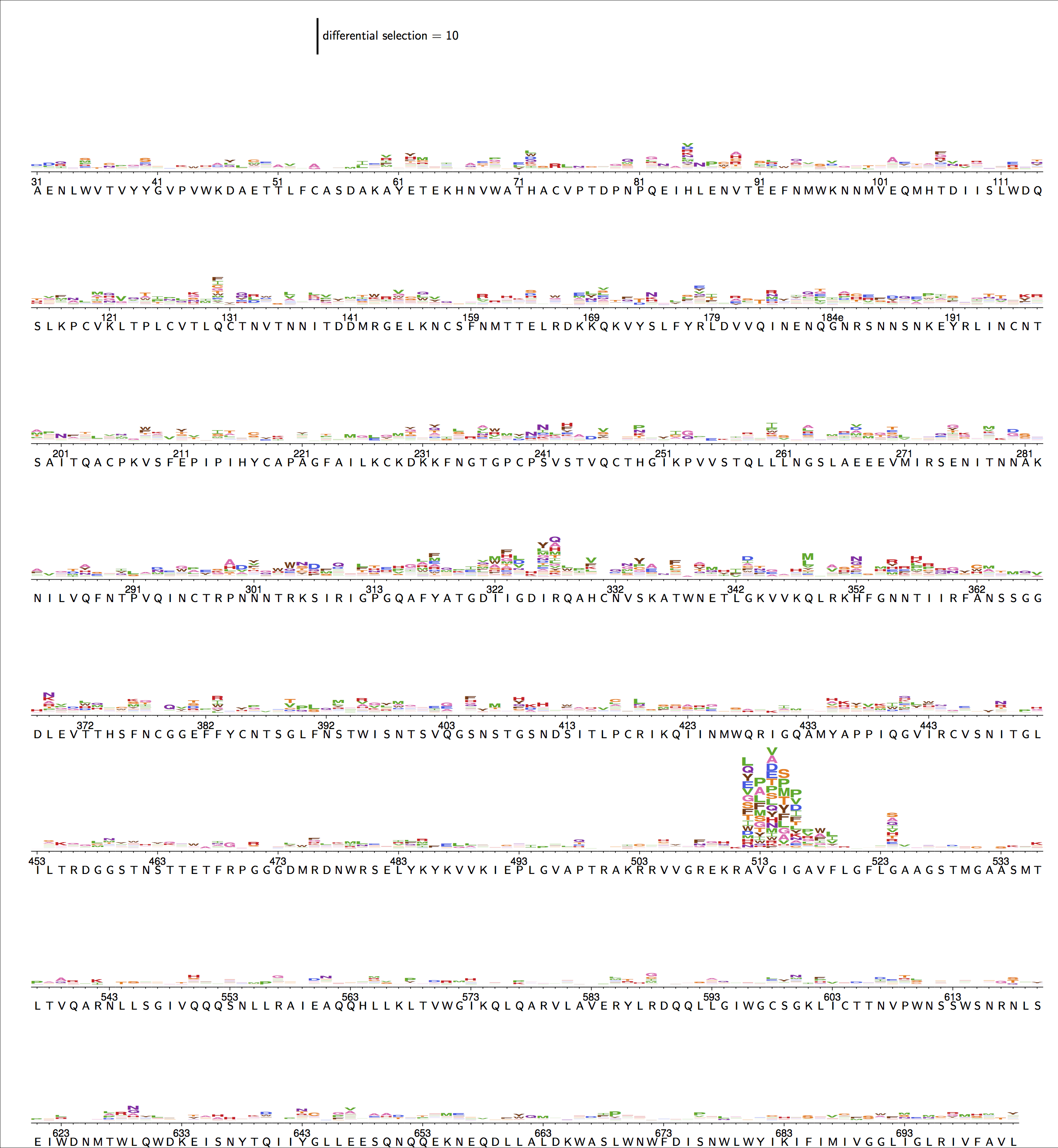

Supplement: S4 Fig — As described for S2 Fig, but for vFP20.01. (TIFF) [file ppat.1007159.s004.tiff]

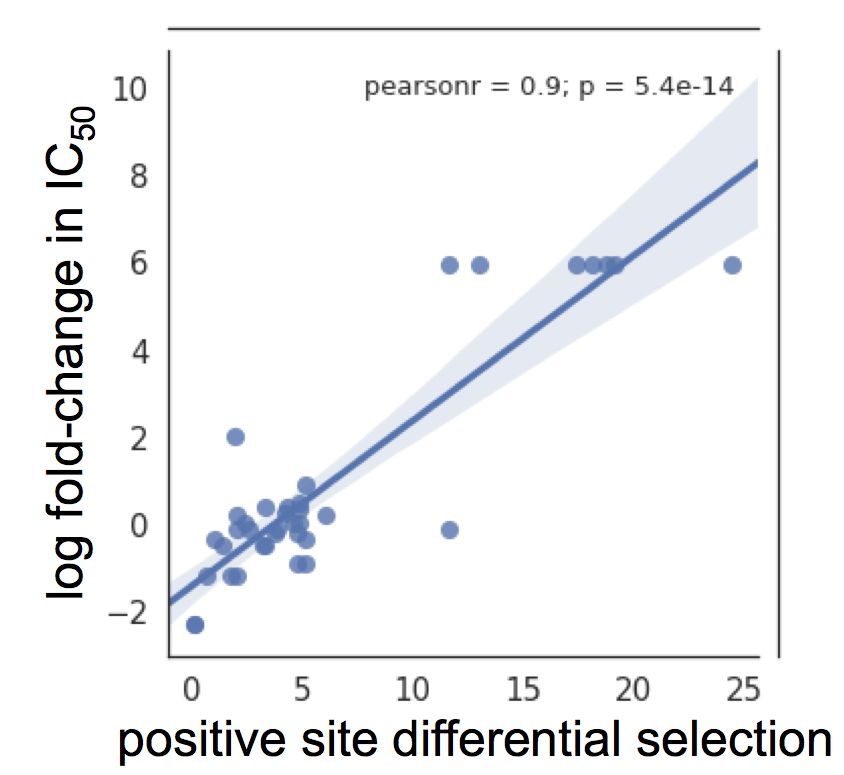

Supplement: S5 Fig — A. Correlation between the positive differential selection at a site and the log fold change in IC50 from a BG505 pseudovirus bearing a point mutant at that site (TZM-bl data from Kong et al (2016) [5], as described in S2 Fig). Note that the TZM-bl data is right censored, and it is possible for a mutation to affect the maximum percent neutralization rather than the IC50. (TIFF) [file ppat.1007159.s005.tiff]

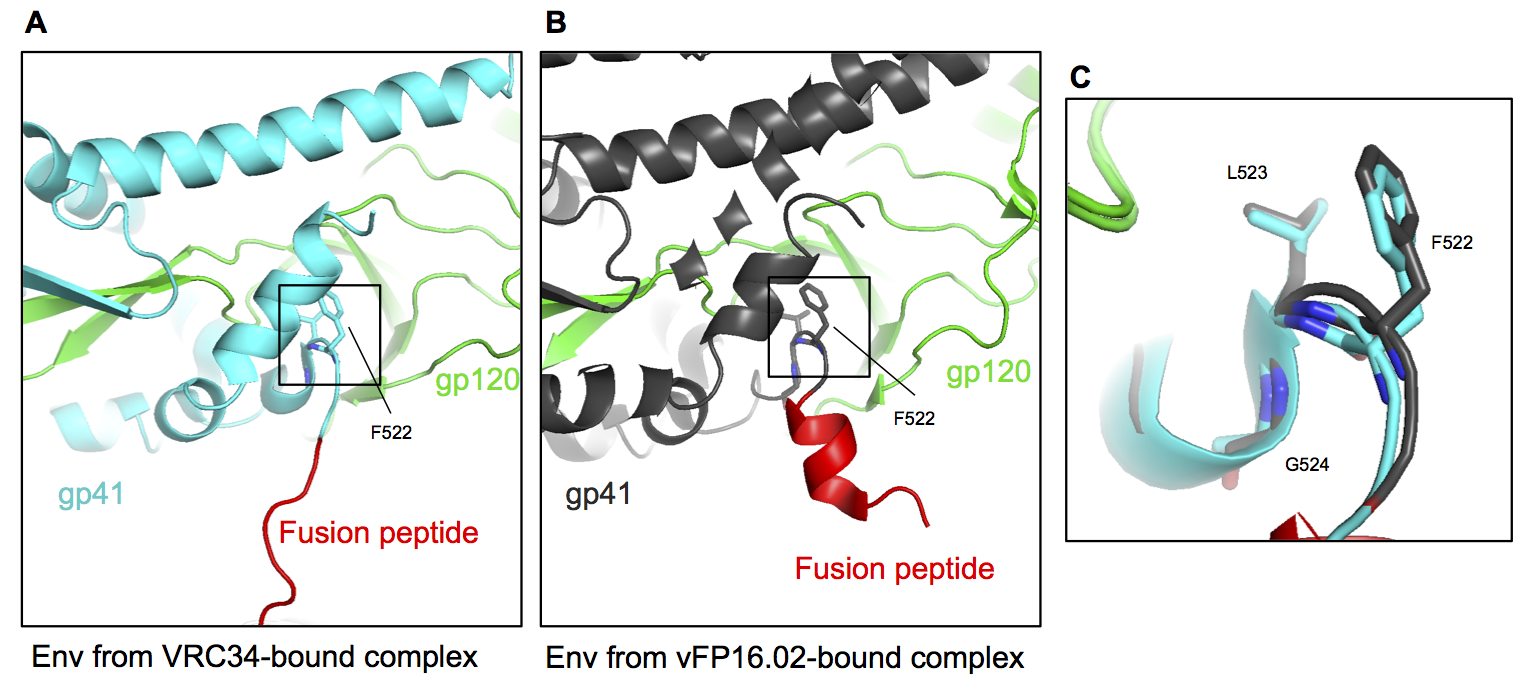

Supplement: S6 Fig — A. Env from VRC34-bound complex with gp41 colored cyan, gp120 colored green and the fusion peptide colored red. Residues 522–524 of gp120 are shown in sticks. B. Same as a, but for Env from vFP16.02-bound complex and gp41 shown in dark gray. C. Overlay of VRC34-bound complex and vFP16.02-bound complex. (TIFF) [file ppat.1007159.s006.tiff]

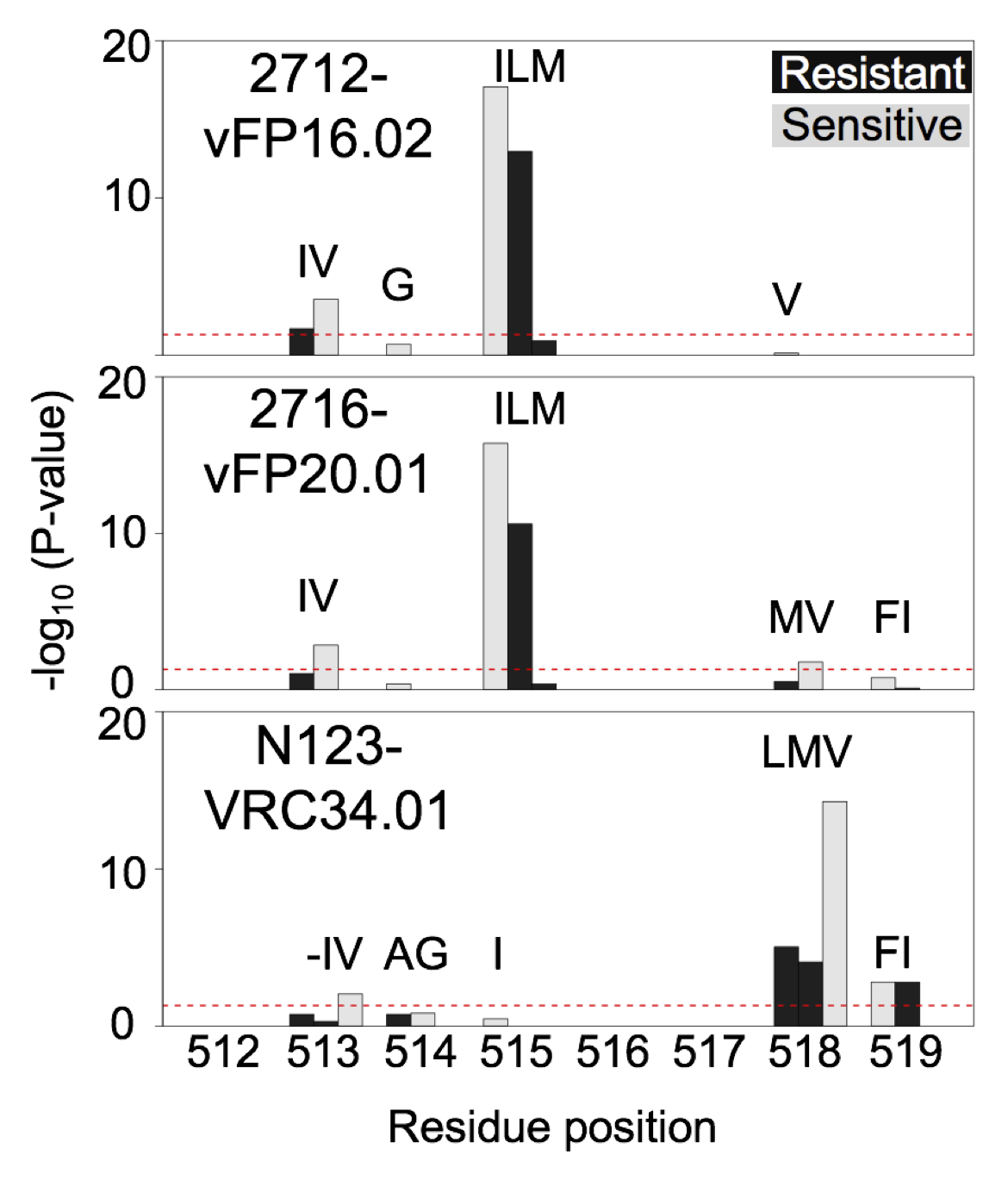

Supplement: S7 Fig — Bar plot shows the P-values of the association (calculated by Fisher’s exact test) between amino acid variation and neutralization at residues 512–519. Black bars highlight amino acid and site combinations that significantly associate with resistance to antibodies, while light-gray bars highlight sensitive ones. Amino acids valine and isoleucine at sites 513 and 515, respectively, are significantly associated with sensitivity, while isoleucine at position 513 and leucine at site 515 are significantly associated with resistance to vFP16.02. In the case of vFP20.01, residues valine, isoleucine and valine at sites 513, 515 and 518, respectively, are associated to sensitivity to vFP20.01, with only leucine at position 515 being significantly associated to resistance. For VRC34, valine at positions 513 and 518 as well as phenylalanine at site 519 are significantly associated to sensitivity to VRC34, while leucine and methionine at site 518, and isoleucine at site 519 are associated with resistance. A dotted red line corresponds to the adjusted P-value threshold of 0.05. (TIFF) [file ppat.1007159.s007.tiff]

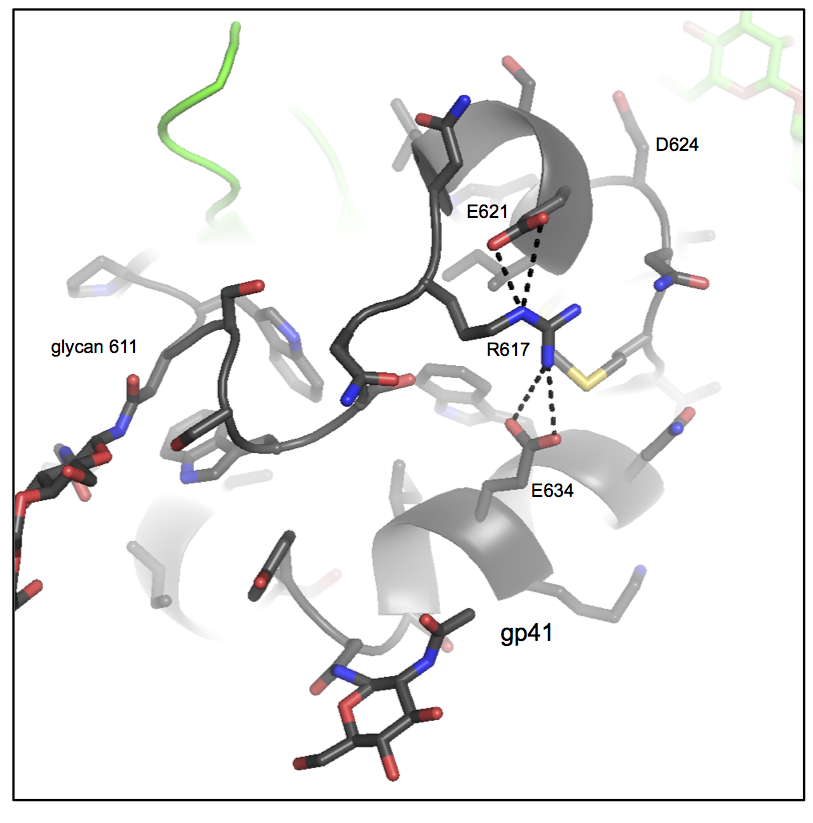

Supplement: S8 Fig — Zoom-in of gp41 region showing a surface salt bridge network formed by residues E621, R617 and E634. (TIFF) [file ppat.1007159.s008.tiff]

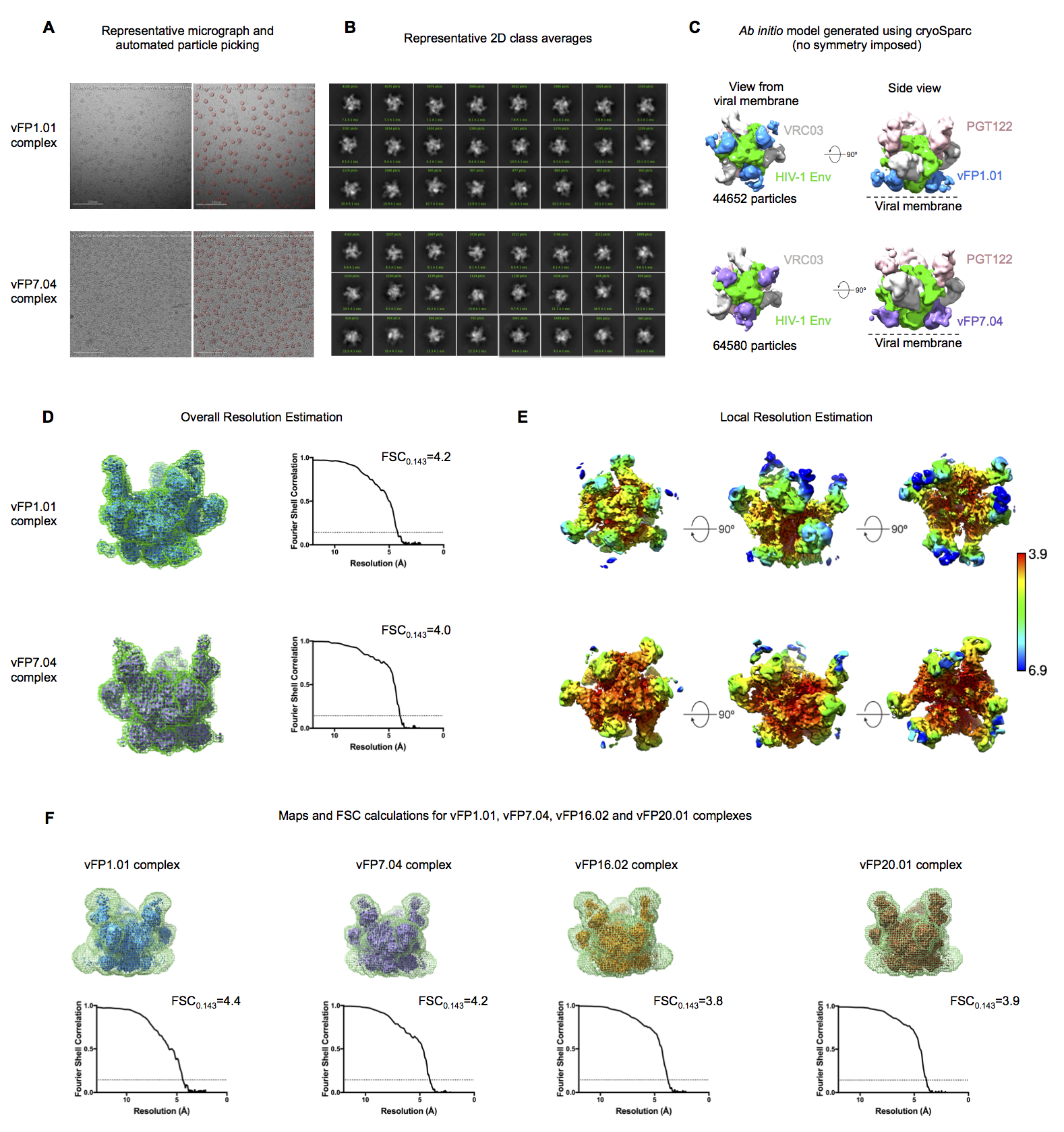

Supplement: S9 Fig — A. Representative micrograph (left) with particles picked with DogPicker shown in red circles (right). B. Representative 2D class averages. C. Ab initio models generated using cryoSparc. D. Refined map in blue (for vFP1.01 complex) and purple (for vFP7.04 complex) with the mask used in RELION postprocessing shown in green. FSC plots and resolution values reported by RELION according to the gold standard FSC0.143 criterion (FSC0.143 shown as dotted line. E. Local resolution estimation. F. For density quantifications we used maps that were postprocessed using expanded solvent masks that encompassed the entire complex, including the more disordered constant domains. The FSC plots and overall resolutions of these maps are shown. The maps were then low-pass filtered to 4.5 Å for the density quantifications. (TIFF) [file ppat.1007159.s009.tiff]

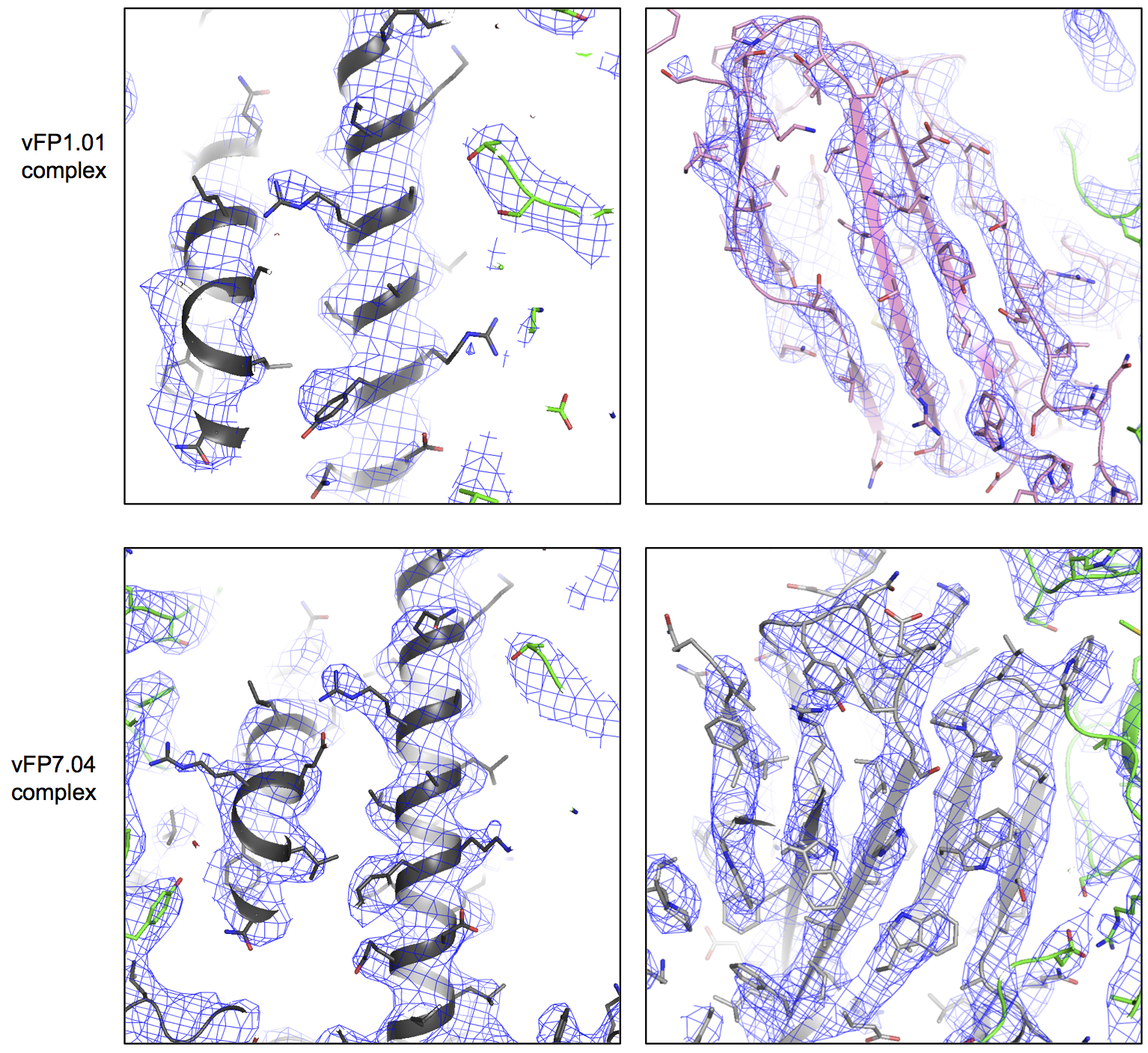

Supplement: S10 Fig — Electron density is shown as a blue mesh and the fitted model is shown as a cartoon with residue side-chains shown as sticks. (TIFF) [file ppat.1007159.s010.tiff]

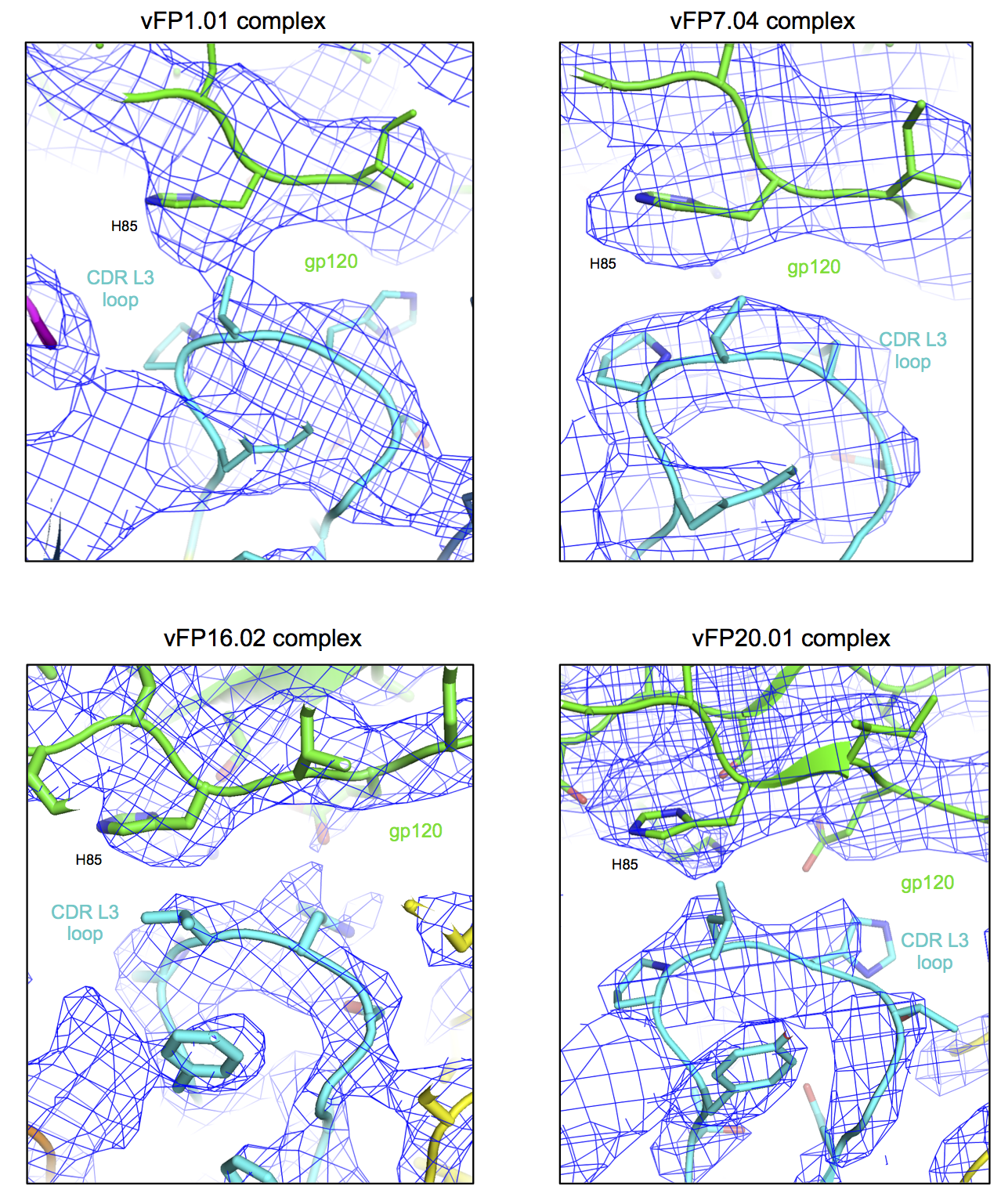

Supplement: S11 Fig — (TIFF) [file ppat.1007159.s011.tiff]

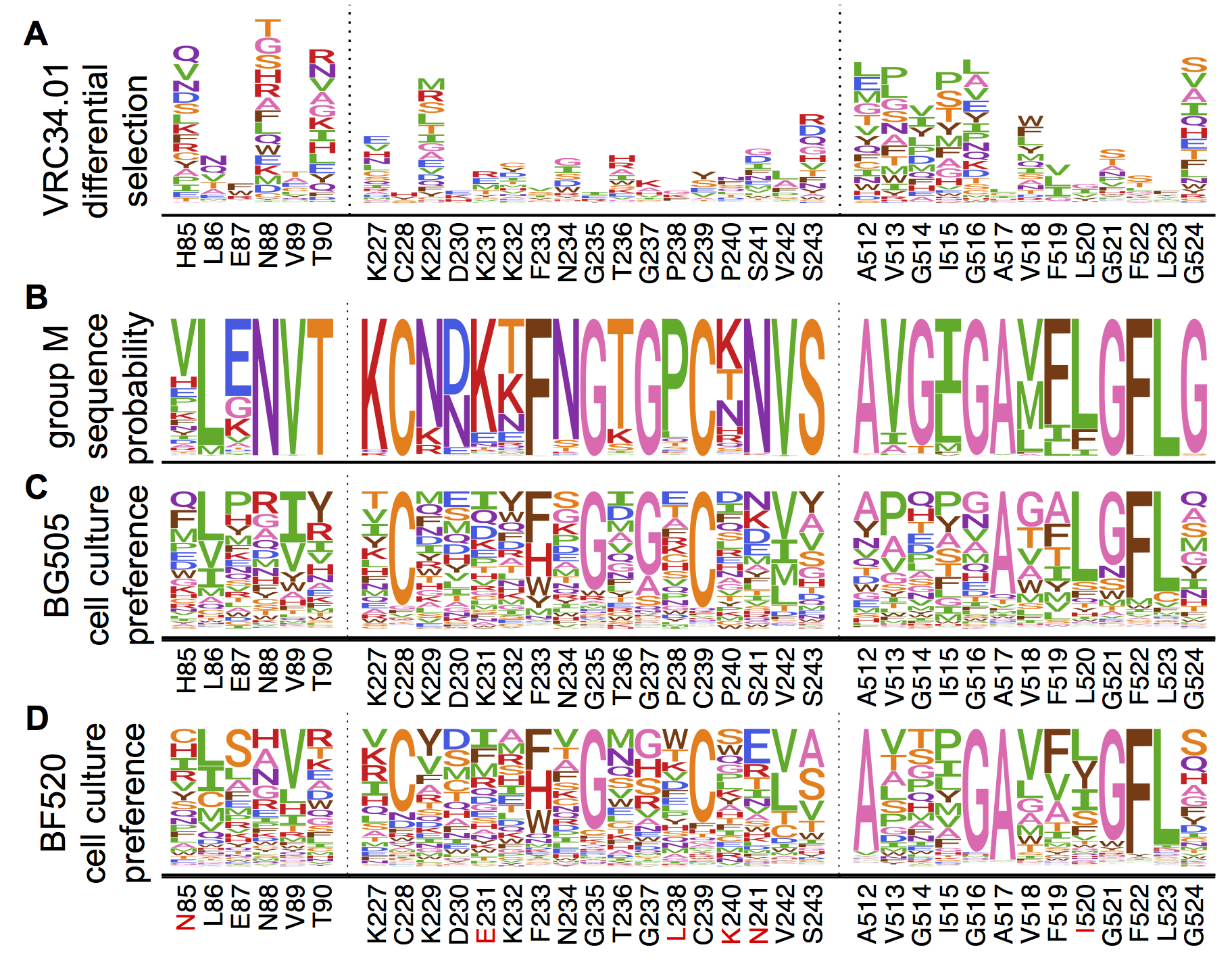

Supplement: S12 Fig — A. The VRC34.01 escape profile is shown as in Fig 2A. B. The amino-acid frequencies in nature, calculated from the group M LANL Web Alignment. C. The BG505 amino-acid preferences under selection for viral replication in cell culture, as measured in Haddox et al 2018 [14]. Briefly, the height of each amino acid corresponds to how well tolerated that amino acid is for viral replication in cell culture. D. The BF520 amino-acid preferences under selection for viral replication in cell culture, as measured in Haddox et al 2018 [14]. For BF520, wildtype amino acids that differ from BG505 are colored red. (TIFF) [file ppat.1007159.s012.tiff]

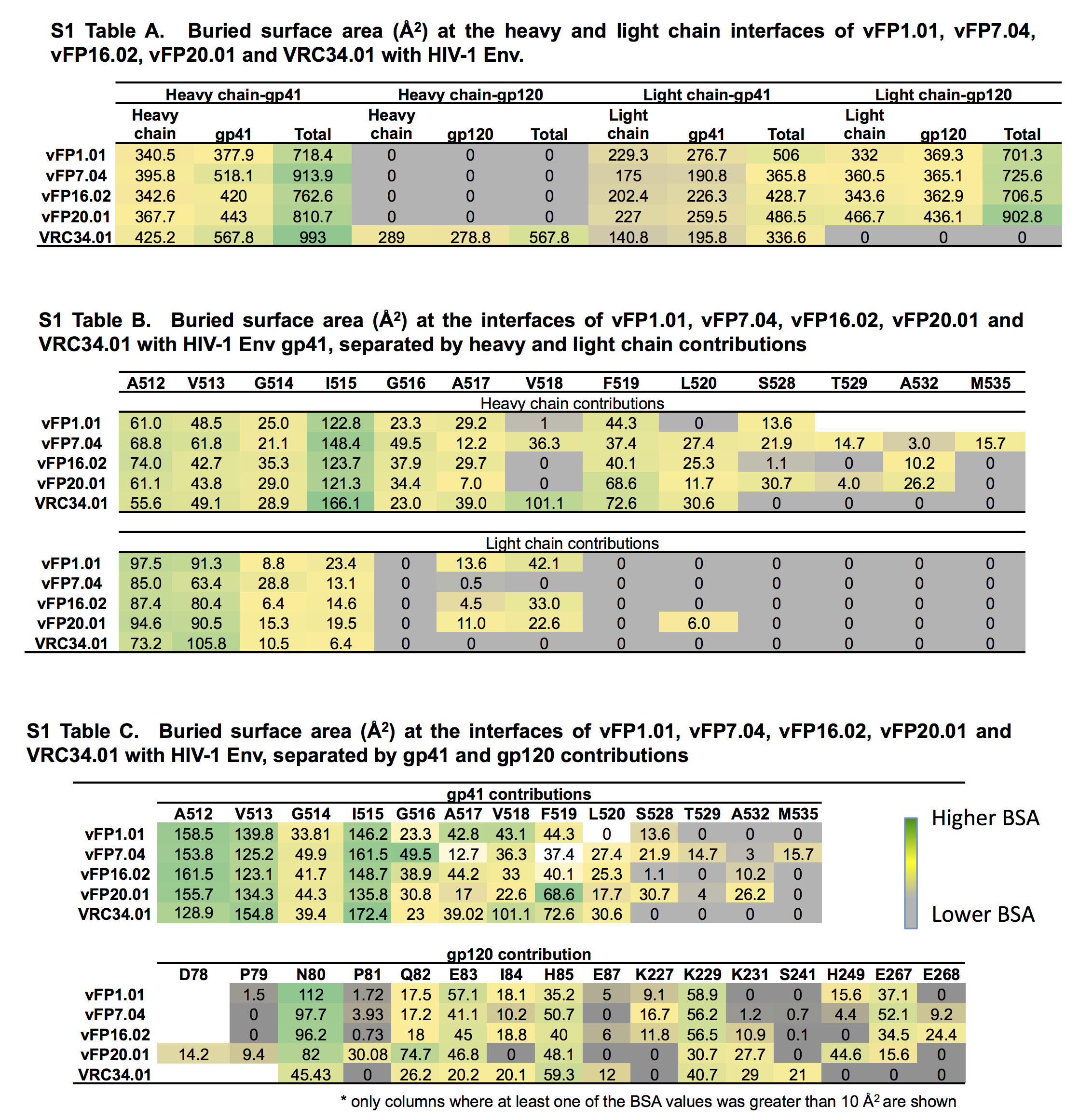

Supplement: S1 Table — (TIFF) [file ppat.1007159.s013.tiff]
