# Supplementary material for: Complete functional mapping of infection- and vaccine-elicited antibodies against the fusion peptide of HIV
Source: PLoS Pathog. 2018 Jul 5;14(7):e1007159. doi: 10.1371/journal.ppat.1007159 (PMC6049957; doi:10.1371/journal.ppat.1007159)
Supplement: S2 Table — (DOC) [file ppat.1007159.s014.doc]

**Table S2 | Cryo-EM data collection, refinement and validation statistics**

|  | **vFP1.01-DS-SOSIP-VRC03-PGT122** | **vFP7.04-DS-SOSIP-VRC03-PGT122** |
| --- | --- | --- |
|  | **EMD-7622; PDB 6CUF** | **EMD-7621; PDB 6CUE** |
| **Data collection and processing** |  |  |
| Magnification | 130000 | 105000 |
| Voltage (kV) | 300 | 300 |
| Electron exposure (e–/Å^2^) | 70.28 | 53.11 |
| Defocus range (μm) | -1.5 - -3.0 | -1.5 - -3.0 |
| Pixel size (Å) | 1.06 | 1.1 |
| Symmetry imposed | C3 | C3 |
| Initial particle images (no.) | 108758 | 152206 |
| Final particle images (no.) | 44652 | 64580 |
| Map resolution (Å)  FSC threshold | 4.2  0.143 | 4.0  0.143 |
| Map resolution range (Å) | 3.9-9.4 | 3.8-8.8 |
|  |  |  |
| **Refinement** |  |  |
| Initial model used (PDB code) | 6CDI | 6CDI |
| Model resolution (Å)  FSC threshold | 4.4  0.5 | 4.1  0.5 |
| Model resolution range (Å) | 4.0-6.0 | 3.9-6.0 |
| Map sharpening *B* factor (Å^2^) | -92.99 | -87.94 |
| Model composition  Non-hydrogen atoms  Protein residues | 3843 | 3849 |
| R.m.s. deviations  Bond lengths (Å)  Bond angles (°) | 0.008  1.770 | 0.007  0.992 |
| Validation  MolProbity score  Clashscore  Poor rotamers (%) | 1.89  8.0  0.81 | 1.72  5.0  0.21 |
| Ramachandran plot  Favored (%)  Allowed (%)  Disallowed (%) | 92.59  7.33  0.08 | 92.57  7.43  0.00 |
