# Supplementary material for: Complete functional mapping of infection- and vaccine-elicited antibodies against the fusion peptide of HIV
Source: PLoS Pathog. 2018 Jul 5;14(7):e1007159. doi: 10.1371/journal.ppat.1007159 (PMC6049957; doi:10.1371/journal.ppat.1007159)
Supplement: S2 File — This zipped file contains the entire mutational antigenic profiling analysis. There is an html document of the completed analysis, an executable IPython notebook, and all of the necessary input data to execute the analysis. (GZ) [file ppat.1007159.s016.gz › FileS1/analysis_notebook.html]

analysis\_notebook


# Complete functional mapping of infection- and vaccine-elicited antibodies against the fusion peptide of HIV¶

### Adam S. Dingens, Hugh K. Haddox, Julie Overbaugh, and Jesse D. Bloom¶

### In collaboration with the Kwong group at the VRC¶

Experiments analyzed here were performed by Adam Dingens in the Bloom lab and Overbaugh lab in the summer/autumn of 2017.

Here, we use mutational antigenic profiling to comprehensively map escape from `N123-VRC34.01`, `2712-vFP16.02`, and `2716-vFP20.01` (subsequently named `VRC34`, `FP16-02`, and `FP20-01` in this iPython notebook) using **BG505.T332N** mutant Env virus libraries.The generation and characterization of the BG505 mutant virus libraires is described in detail in Haddox, Dingens et al. bioRxiv 2017.

Dingens et al. Cell Host & Microbe 2017 details mutational antigenic profiling of PGT151 using BF520 Env mutant virus libraries.

Here, we use dms\_tools2 to analyze the data. This notebook processes the Illumina deep sequencing data, and then analyzes the selection in the context of the antibody.

The barcoded subamplicon Illumina sequencing approach is described in detail here, and the computation of `differential selection` is described here.

## Import `Python` modules, define widely used functions¶

Set `use_existing` to `yes` below if you want to use any existing output, and `no` if you want to re-generate all output.

In [1]:

```
import os
import glob
import pandas as pd
from IPython.display import display, HTML
import dms_tools2
import dms_tools2.plot
import dms_tools2.sra
import dms_tools2.utils
import dms_tools2.diffsel
from dms_tools2.ipython_utils import showPDF
import rpy2
import rpy2.robjects 
import dms_tools2.rplot

import numpy as np
import pylab as plt
from colour import Color
print("Using dms_tools2 version {0}".format(dms_tools2.__version__))
# results will go in this directory
resultsdir = './results/' 
if not os.path.isdir(resultsdir):
    os.mkdir(resultsdir)
    
# CPUs to use, -1 means all available
ncpus = 14

# do we use existing results or generate everything new?
use_existing = 'yes'
```

```
/usr/lib/python3.4/importlib/_bootstrap.py:321: FutureWarning: The pandas.core.datetools module is deprecated and will be removed in a future version. Please use the pandas.tseries module instead.
  return f(*args, **kwds)
```

```
Using dms_tools2 version 2.2.dev4
```

## Download the sequencing data from the Sequence Read Archive¶

Here we download sequencing data for each sample from the Sequence Read Archive. Sample names specificy if they are mutant (mut) or wildtype (wt), as well as virus (virus) or DNA (DNA). The *#* is which replicate (1 and 3). Note that we use BG505 mutant virus libraries #1 and #3, a numbering notation that matches the description of these libraries. In the figures and text, we name `replicate 3` as `replicate 2` for simplicity. If a sample is antibody selected, it also has the antibody and `ug/mL` concentration. There is metadata available from the SRA, and the dictionary below also specifies the accession number of each sample.

They reads were submitted as SRA submission SUB3391371 with BioSample numbers SAMN08275788-SAMN08275801 with SRA accession numbers `SRR6429862-SRR6429875` on Dec 23, 2017. The BioProject ID is `PRJNA427906`.

We download these files using the dms\_tools2.sra.fastqFromSRA function from the dms\_tools2 Python API. Note that the call to this function below uses two external programs that are not part of dms\_tools2, and which you therefore must install externally on the computer that you are using:

1. The fastq-dump program from the SRA Toolkit. If you do not already have this toolkit installed, you will need to install a relatively recent version.
2. The Aspera Connect program for rapid downloads. You need both the executable ascp and an Aspera Key file. Installing Aspera Connect and a key can be somewhat complex, so if you do not want to do this then just set aspera=None in the command below and fastq-dump will do the downloads (albeit more slowly).

In [2]:

```
samples = pd.DataFrame.from_records(
        [('BG505_mut-DNA_1', 'SRR6429865'),
         ('BG505_mut-DNA_3', 'SRR6429864'),
         ('BG505_mut-virus_1_FP16-02_500ug', 'SRR6429867'),
         ('BG505_mut-virus_1_FP20-01_500ug', 'SRR6429866'),
         ('BG505_mut-virus_1', 'SRR6429869'),
         ('BG505_mut-virus_1_VRC34_33ug', 'SRR6429868'),
         ('BG505_mut-virus_3_FP16-02_500ug', 'SRR6429871'),
         ('BG505_mut-virus_3_FP20-01_500ug', 'SRR6429870'),
         ('BG505_mut-virus_3', 'SRR6429863'),
         ('BG505_mut-virus_3_VRC34_33ug', 'SRR6429862'),
         ('BG505_wt-DNA_1', 'SRR6429875'),
         ('BG505_wt-DNA_3', 'SRR6429874'),
         ('BG505_wt-virus_1', 'SRR6429873'),
         ('BG505_wt-virus_3', 'SRR6429872')],       
        columns=['name', 'run']
        )


fastqdir = 'results/FASTQ_files/'
print("Downloading FASTQ files from the SRA...")
dms_tools2.sra.fastqFromSRA(
        samples=samples,
        fastq_dump='fastq-dump', # valid path to this program on the Hutch server
        fastqdir=fastqdir,
        aspera=(
            '/app/aspera-connect/3.5.1/bin/ascp', # valid path to ascp on Hutch server
            '/app/aspera-connect/3.5.1/etc/asperaweb_id_dsa.openssh' # Aspera key on Hutch server
            ),
        )
print("Here are the names of the downloaded files now found in {0}".format(fastqdir))
display(HTML(samples.to_html(index=False)))
```

```
Downloading FASTQ files from the SRA...
Here are the names of the downloaded files now found in results/FASTQ_files/
```

| name | run | R1 | R2 |
| --- | --- | --- | --- |
| BG505\_mut-DNA\_1 | SRR6429865 | BG505\_mut-DNA\_1\_R1.fastq.gz | BG505\_mut-DNA\_1\_R2.fastq.gz |
| BG505\_mut-DNA\_3 | SRR6429864 | BG505\_mut-DNA\_3\_R1.fastq.gz | BG505\_mut-DNA\_3\_R2.fastq.gz |
| BG505\_mut-virus\_1\_FP16-02\_500ug | SRR6429867 | BG505\_mut-virus\_1\_FP16-02\_500ug\_R1.fastq.gz | BG505\_mut-virus\_1\_FP16-02\_500ug\_R2.fastq.gz |
| BG505\_mut-virus\_1\_FP20-01\_500ug | SRR6429866 | BG505\_mut-virus\_1\_FP20-01\_500ug\_R1.fastq.gz | BG505\_mut-virus\_1\_FP20-01\_500ug\_R2.fastq.gz |
| BG505\_mut-virus\_1 | SRR6429869 | BG505\_mut-virus\_1\_R1.fastq.gz | BG505\_mut-virus\_1\_R2.fastq.gz |
| BG505\_mut-virus\_1\_VRC34\_33ug | SRR6429868 | BG505\_mut-virus\_1\_VRC34\_33ug\_R1.fastq.gz | BG505\_mut-virus\_1\_VRC34\_33ug\_R2.fastq.gz |
| BG505\_mut-virus\_3\_FP16-02\_500ug | SRR6429871 | BG505\_mut-virus\_3\_FP16-02\_500ug\_R1.fastq.gz | BG505\_mut-virus\_3\_FP16-02\_500ug\_R2.fastq.gz |
| BG505\_mut-virus\_3\_FP20-01\_500ug | SRR6429870 | BG505\_mut-virus\_3\_FP20-01\_500ug\_R1.fastq.gz | BG505\_mut-virus\_3\_FP20-01\_500ug\_R2.fastq.gz |
| BG505\_mut-virus\_3 | SRR6429863 | BG505\_mut-virus\_3\_R1.fastq.gz | BG505\_mut-virus\_3\_R2.fastq.gz |
| BG505\_mut-virus\_3\_VRC34\_33ug | SRR6429862 | BG505\_mut-virus\_3\_VRC34\_33ug\_R1.fastq.gz | BG505\_mut-virus\_3\_VRC34\_33ug\_R2.fastq.gz |
| BG505\_wt-DNA\_1 | SRR6429875 | BG505\_wt-DNA\_1\_R1.fastq.gz | BG505\_wt-DNA\_1\_R2.fastq.gz |
| BG505\_wt-DNA\_3 | SRR6429874 | BG505\_wt-DNA\_3\_R1.fastq.gz | BG505\_wt-DNA\_3\_R2.fastq.gz |
| BG505\_wt-virus\_1 | SRR6429873 | BG505\_wt-virus\_1\_R1.fastq.gz | BG505\_wt-virus\_1\_R2.fastq.gz |
| BG505\_wt-virus\_3 | SRR6429872 | BG505\_wt-virus\_3\_R1.fastq.gz | BG505\_wt-virus\_3\_R2.fastq.gz |

## Define samples from FASTQ\_files¶

In [4]:

```
fastqdir = './results/FASTQ_files/'
R1fastqfilelist = (glob.glob("./results/FASTQ_files/*R1.fastq.gz"))
R1fastqfilelist_df = pd.DataFrame(R1fastqfilelist)
R1fastqfilelist_df.columns = ['R1']
R1fastqfilelist_df.replace('./results/FASTQ_files/', '', regex=True, inplace=True)
R1fastqfilelist_df['name'] = R1fastqfilelist_df['R1']
R1fastqfilelist_df['name'].replace('_R1.fastq.gz', '', regex=True, inplace=True)
R1fastqfilelist_df['name'].replace('_', '-', regex=True, inplace=True)
R1fastqfilelist_df['name'].replace('\.', '-', regex=True, inplace=True)
R1fastqfilelist_df['name'].replace('BG505-', '', regex=True, inplace=True)

#R1fastqfilelist_df
display(HTML(R1fastqfilelist_df.to_html(index=False)))
```

| R1 | name |
| --- | --- |
| BG505\_mut-virus\_1\_R1.fastq.gz | mut-virus-1 |
| BG505\_mut-virus\_3\_FP20-01\_500ug\_R1.fastq.gz | mut-virus-3-FP20-01-500ug |
| BG505\_mut-virus\_3\_VRC34\_33ug\_R1.fastq.gz | mut-virus-3-VRC34-33ug |
| BG505\_wt-virus\_3\_R1.fastq.gz | wt-virus-3 |
| BG505\_mut-virus\_3\_R1.fastq.gz | mut-virus-3 |
| BG505\_mut-DNA\_3\_R1.fastq.gz | mut-DNA-3 |
| BG505\_mut-virus\_1\_VRC34\_33ug\_R1.fastq.gz | mut-virus-1-VRC34-33ug |
| BG505\_wt-DNA\_1\_R1.fastq.gz | wt-DNA-1 |
| BG505\_mut-virus\_1\_FP16-02\_500ug\_R1.fastq.gz | mut-virus-1-FP16-02-500ug |
| BG505\_wt-DNA\_3\_R1.fastq.gz | wt-DNA-3 |
| BG505\_mut-virus\_3\_FP16-02\_500ug\_R1.fastq.gz | mut-virus-3-FP16-02-500ug |
| BG505\_wt-virus\_1\_R1.fastq.gz | wt-virus-1 |
| BG505\_mut-virus\_1\_FP20-01\_500ug\_R1.fastq.gz | mut-virus-1-FP20-01-500ug |
| BG505\_mut-DNA\_1\_R1.fastq.gz | mut-DNA-1 |

## Process the FASTQ files to count the mutations for each sample¶

We used barcoded-subamplicon sequencing to obtain high accuracy during the Illumina deep sequencing. We therefore use the dms2\_batch\_bcsubamp program to analyze these data.

Running that program requires specifying a `--batchfile` that lists the samples, a wildtype `--refseq` to which we make alignments, and --alignspecs that tell us where the subamplicons should align.
The batch file that we specify is printed by the cell below. The alignment specs need to be exactly correct for the subamplicons to align. We also do some trimming of the reads using the `--R1trim` and `--R2trim` parameters.

The wildtype sequence of the BG505.W6.C2.T332N Env used in this experiment is in the file ./data/BG505.W6.C2.T332N\_env.fasta.
This sequence is based on GenBank accession number DQ208458.1, with the introduced T332N mutation to knock in the glycan commonly targeted by V3/N332 class bnAbs.

In [16]:

```
refseq = './data/BG505.W6.C2.T332N_env.fasta'
#sitestoinclude = "./data/BG505_sitestoinclude.csv"
sitemask = "./data/sitemask_BG505.txt"

# define subamplicon alignment specifications
alignspecs = ' '.join(['87,375,39,36', 
                       '376,666,36,39',
                       '663,954,33,41',
                       '955,1228,33,37',
                       '1228,1527,34,35',
                       '1527,1815,32,39',
                       '1816,2098,36,41'])

# counts and alignments placed in this directory
countsdir = os.path.join(resultsdir, 'codoncounts')
if not os.path.isdir(countsdir):
    os.mkdir(countsdir)
    
# write sample information to a batch file for dms2_batch_bcsubamplicons
countsbatchfile = os.path.join(countsdir, 'batch.csv')
print("Here is the batch file that we write to CSV format to use as input:")
display(HTML(R1fastqfilelist_df[['name', 'R1']].to_html(index=False)))
R1fastqfilelist_df[['name', 'R1']].to_csv(countsbatchfile, index=False)

print('\nNow running dms2_batch_bcsubamp...')
log = !dms2_batch_bcsubamp \
        --batchfile {countsbatchfile} \
        --refseq {refseq} \
        --alignspecs {alignspecs} \
        --outdir {countsdir} \
        --summaryprefix summary \
        --R1trim 200 \
        --R2trim 170 \
        --minq 15 \
        --fastqdir {fastqdir} \
        --ncpus {ncpus} \
        --sitemask {sitemask} \
        --use_existing {use_existing} 
print("Completed dms2_batch_bcsubamp.")
```

```
Here is the batch file that we write to CSV format to use as input:
```

| name | R1 |
| --- | --- |
| mut-virus-1 | BG505\_mut-virus\_1\_R1.fastq.gz |
| mut-virus-3-FP20-01-500ug | BG505\_mut-virus\_3\_FP20-01\_500ug\_R1.fastq.gz |
| mut-virus-3-VRC34-33ug | BG505\_mut-virus\_3\_VRC34\_33ug\_R1.fastq.gz |
| wt-virus-3 | BG505\_wt-virus\_3\_R1.fastq.gz |
| mut-virus-3 | BG505\_mut-virus\_3\_R1.fastq.gz |
| mut-DNA-3 | BG505\_mut-DNA\_3\_R1.fastq.gz |
| mut-virus-1-VRC34-33ug | BG505\_mut-virus\_1\_VRC34\_33ug\_R1.fastq.gz |
| wt-DNA-1 | BG505\_wt-DNA\_1\_R1.fastq.gz |
| mut-virus-1-FP16-02-500ug | BG505\_mut-virus\_1\_FP16-02\_500ug\_R1.fastq.gz |
| wt-DNA-3 | BG505\_wt-DNA\_3\_R1.fastq.gz |
| mut-virus-3-FP16-02-500ug | BG505\_mut-virus\_3\_FP16-02\_500ug\_R1.fastq.gz |
| wt-virus-1 | BG505\_wt-virus\_1\_R1.fastq.gz |
| mut-virus-1-FP20-01-500ug | BG505\_mut-virus\_1\_FP20-01\_500ug\_R1.fastq.gz |
| mut-DNA-1 | BG505\_mut-DNA\_1\_R1.fastq.gz |

```
Now running dms2_batch_bcsubamp...
Completed dms2_batch_bcsubamp.
```

Now we look at the summary plots created by `dms2_batch_bcsubamp`.
All of these files are found in the directory specified by --outdir, and with the prefix specified by summaryprefix.
So we define them using this plot prefix plus the suffix for each plot.
Note that these files all refer to sites in sequential 1, 2, ... numbering of the BG505 sequence.

In [17]:

```
countsplotprefix = os.path.join(countsdir, 'summary')
```

The `*_readstats.pdf` plot below shows the statistics on the reads. This plot shows that most of the reads were retained, and a small fraction discarded because of low-quality barcodes. None failed the Illumina filter as those were already filtered out those when we downloaded from the SRA.

In [18]:

```
showPDF(countsplotprefix + '_readstats.pdf', width=600)
```

The `*_readsperbc.pdf` plot below shows how many times different barcodes were observed for each sample. Barcodes need to be observed multiple times to be useful for barcoded-subamplicon sequencing error correction.

In [19]:

```
showPDF(countsplotprefix + '_readsperbc.pdf')
```

The `*_bcstats.pdf` plot below shows statistics on the barcodes. Some of the barcodes had to be discarded because they had too few reads (these are the single-read barcodes in the plot above), a small fraction with adequate reads were not alignable, and the rest aligned to the Env gene properly.
This plot and the one above suggest that probably could have gotten additional depth by sequencing more, since then we would have had more barcodes with multiple reads.

In [20]:

```
showPDF(countsplotprefix + '_bcstats.pdf', width=600)
```

The `*_depth.pdf` plot below shows the depth (number of called codons) at each site in the gene.
For most of the samples, the depth across the gene is fairly uniform, indicating that the subamplicons were pooled fairly evenly.  
Note that some samples (in particular the mock samples) were intentionally sequenced to higher depth than the antibody-selected samples, as we expect to see more diversity in the mock samples.

In [21]:

```
showPDF(countsplotprefix + '_depth.pdf')
```

The `*_mutfreq.pdf` plot below shows the per-codon frequency of mutations at each site.
For each antibody-selected sample, we see a few sites of clear peaks in mutation frequency.
These peaks tend to occur at the same sites in different replicates, and so presumably represent the sites where antibody-escape mutations are selected.
There are no such peaks for the mock sample since there is no antibody selection to favor specific mutations.
Note also that the gene was not mutagenized or sequenced in the signal peptide or cytoplasmic tail.

In [22]:

```
showPDF(countsplotprefix + '_mutfreq.pdf')
```

The `*_codonmuttypes.pdf` plot below shows the per-codon frequency of nonsynonymous, synonymous, and stop codon mutations across the entire gene. For the antibody selected samples, we see an overall increase in the per-codon mutation frequency due to very strong selection for variants with escape mutations.

In [23]:

```
showPDF(countsplotprefix + '_codonmuttypes.pdf', width=600)
```

The `*_codonntchanges.pdf` plot below shows same data as above but categorizes codon mutations by the number of nucleotides that are changed (e.g., ATG to AAG changes 1 nucleotide, ATG to AAC changes 2 nucleotides, and ATG to CAC changes 3 nucleotides).

In [24]:

```
showPDF(countsplotprefix + '_codonntchanges.pdf', width=600)
```

The `*_singlentchanges.pdf` plot below shows the frequency of each type of nucleotide change among only codon mutations with one nucleotide change. This plot is mostly useful to check if there is a large bias in which mutations appear. In particular, if you are getting oxidative damage (which causes G to T mutations) during the library preparation process, you will see a large excess of C to A or G to T mutations (or both). There is not much oxidative damage in the samples plotted below, which mostly have a fairly even distribution of nucleotide changes.

We do see that transitions (G <-> A and C <-> T) are a bit more common than most of the other types of mutations (all of which are transversions). This is expected, since PCR based sources of mutations are expected to preferentially introduce transitions.

This plot would also be important to examine any sign of APOBEC hypermutation (also G <-> A, with preferences for specific motifs) occuring in the HIV genome. One of the reasons we selected the SupT1.R5 cell line was because there is very little of the APOBEC proteins expressed in a subclone of SupT1 cells. In the viral samples, there does appear to be an increase in G to A and C to T mutations. However, we passaged WT virus in these cells and confirmed there were not signs of extensive APOBEC hypermutation in hotspot motifs via deep sequencing of env after passaging. Thus, we do not think there is extensive APOBEC hypermutation in our data, and this increase certain types of mutations in the viral samples is likely due to bias in the viral reverse transcription.

In [25]:

```
showPDF(countsplotprefix + '_singlentchanges.pdf', width=600)
```

## Renumber codon counts to HXB2 numbering¶

The standard numbering scheme for HIV Env is the HXB2 numbering scheme.
The file ./data/BG505\_to\_HXB2\_numbering.txt gives the mapping from sequential 1, 2, ... numbering of the BG505 protein sequence to the HXB2 numbering scheme.
This file was generated by aligning the HXB2 sequence taken from Genbank with the BG505C2.T332N sequence at the protein sequence level.
Insertions relative to HXB2 are given letter suffixes as described here.

Additionally, not all residues in BG505 Env were mutagenized.
The N-terminal signal peptide and the C-terminal cytoplasmic tail were excluded because they seem likely to affect expression level.
These sites are not listed in the renumbering file, and so are dropped when we do the re-numbering.

To do the re-numbering, we use the dms\_tools2.utils.renumberSites function from the dms\_tools Python API to create a new directory that contains all of the re-numbered files with the same name as in the original codon counts directory produced above.

In [26]:

```
renumberedcountsdir = os.path.join(resultsdir, 'renumberedcounts')
```

In [27]:

```
renumberfile = './data/BG505_to_HXB2_numbering.txt'

# renumbered counts will go here
renumberedcountsdir = os.path.join(resultsdir, 'renumberedcounts')

# counts files to renumber
countsfiles = glob.glob('{0}/*codoncounts.csv'.format(countsdir))

dms_tools2.utils.renumberSites(renumberfile, countsfiles, missing='drop', 
        outdir=renumberedcountsdir)
```

## Compute the differential selection and correlations between replicates¶

Now we compute the differential selection for each replicate, using the *wt-DNA* controls to estimate the error rates.
We also compute the average differential selection across replicates.
Finally, we look at correlations between pairs of replicates in mutation and site differential selection, restricting to only positive differential selection.
We first create a batch file to use with dms2\_batch\_diffsel.
Note we make the **group** argument the antibody, the **name** argument the replicate, and assign the **sel**, **mock**, and **err** arguments based on the names used for the batch file when generating the counts files above with `dms2_batch_bcsubamp`. Here, we are using sequencing of **wt-DNA** as an error control.

By grouping replicates for the same antibody in the batch file, we tell dms2\_batch\_diffsel to analyze these together and take their mean and median.

This csv file contains the fraction remaining infectivity for each antibody selected sample, as quatified using pol qPCR and computed based on a standard curve of infecting cells with dilutions of mutant virus (library and experiment specific), with dilutiuons ranging from 0.1 to .0001.

Finally, we look at correlations between pairs of replicates for both the `mutdiffsel` and `sitediffsel`.

In [28]:

```
# put diffsel values here
diffseldir = os.path.join(resultsdir, 'diffsel')
if not os.path.isdir(diffseldir):
    os.mkdir(diffseldir)
    
diffsel_wtDNActrl_dir = os.path.join(diffseldir, 'wtDNA_ctrl')
if not os.path.isdir(diffsel_wtDNActrl_dir):
    os.mkdir(diffsel_wtDNActrl_dir)
```

In [29]:

```
#set up diffselbatch
qPCRdata = pd.read_csv('./data/BG505_FP_Abs_qPCR_master.csv')
qPCRdata["sel"].replace('BG505-', '', regex=True, inplace=True)

codoncountslist = (glob.glob("./results/renumberedcounts/*_codoncounts.csv"))
codoncounts_df = pd.DataFrame(codoncountslist)
codoncounts_df.columns = ['codoncounts']
codoncounts_df["samplename"] = codoncounts_df['codoncounts']
codoncounts_df["samplename"].replace('_codoncounts.csv', '', regex=True, inplace=True)
codoncounts_df["samplename"].replace('./results/renumberedcounts/', '', regex=True, inplace=True)
codoncounts_df["samplename"].replace('_', '-', regex=True, inplace=True)
codoncounts_df["replicate"] = codoncounts_df["samplename"].str.split('-').str.get(2)
codoncounts_df["antibody"] = codoncounts_df["samplename"].str.split('-', expand=True, n=3).get(3)
codoncounts_df
sampledf = codoncounts_df.replace(to_replace='None', value="np.nan").dropna()
sampledf["group"] = sampledf['antibody']
sampledf["sel"] = sampledf["samplename"]
sampledf["err"] = sampledf["replicate"]
sampledf['err'] = 'wt-DNA-' + sampledf['err'].astype(str)
sampledf["mock"] = sampledf["replicate"]
sampledf['mock'] = 'mut-virus-' + sampledf['mock'].astype(str)

sampledf["name"] = "rep-" + sampledf['replicate'].astype(str) 
sampledf["mds_names"] = sampledf['group'].astype(str) + "-" + sampledf['name'].astype(str)


diffselbatch = sampledf[['group','name', "sel", "mock", "err", "mds_names"]]
diffselbatch = diffselbatch.sort_values(by='group')

diffselbatch = pd.merge(diffselbatch, qPCRdata[['sel', 'libfracsurvive']], 
        left_on = 'sel', right_on= 'sel') 
#if we wanted to compute the differential selection with different error controls, we could then start from the diffselbatch datafram. 

diffselbatch_wtDNActrl = diffselbatch.copy()
diffselbatch_wtDNActrl['err'].replace('wt-virus', 'wt-DNA', regex=True, inplace=True)

diffselbatchfile_wtDNActrl = os.path.join(diffsel_wtDNActrl_dir, 'batch.csv')
print("Here is the batch file that we write to CSV format to use as input:")
display(HTML(diffselbatch_wtDNActrl.to_html(index=False)))
diffselbatch_wtDNActrl.to_csv(diffselbatchfile_wtDNActrl, index=False)
```

```
Here is the batch file that we write to CSV format to use as input:
```

| group | name | sel | mock | err | mds\_names | libfracsurvive |
| --- | --- | --- | --- | --- | --- | --- |
| FP16-02-500ug | rep-1 | mut-virus-1-FP16-02-500ug | mut-virus-1 | wt-DNA-1 | FP16-02-500ug-rep-1 | 0.052725 |
| FP16-02-500ug | rep-3 | mut-virus-3-FP16-02-500ug | mut-virus-3 | wt-DNA-3 | FP16-02-500ug-rep-3 | 0.041880 |
| FP20-01-500ug | rep-3 | mut-virus-3-FP20-01-500ug | mut-virus-3 | wt-DNA-3 | FP20-01-500ug-rep-3 | 0.038097 |
| FP20-01-500ug | rep-1 | mut-virus-1-FP20-01-500ug | mut-virus-1 | wt-DNA-1 | FP20-01-500ug-rep-1 | 0.047702 |
| VRC34-33ug | rep-3 | mut-virus-3-VRC34-33ug | mut-virus-3 | wt-DNA-3 | VRC34-33ug-rep-3 | 0.050239 |
| VRC34-33ug | rep-1 | mut-virus-1-VRC34-33ug | mut-virus-1 | wt-DNA-1 | VRC34-33ug-rep-1 | 0.063644 |

In [30]:

```
log = !dms2_batch_diffsel \
        --summaryprefix summary \
        --batchfile {diffselbatchfile_wtDNActrl} \
        --outdir {diffsel_wtDNActrl_dir} \
        --indir {renumberedcountsdir} \
        --use_existing {use_existing}
```

### Visualize the average differential selection¶

First, we will plot the positive, and then total, site differential selection across the length of the gene.

In [ ]:

```
figuredir = "./results/diffsel/Figures/"
if not os.path.isdir(figuredir):
    os.mkdir(figuredir)
```

In [2]:

```
names = ["VRC34.01", "vFP16.02", "vFP20.01"]
diffselfiles = ["./results/diffsel/wtDNA_ctrl/summary_VRC34-33ug-meansitediffsel.csv", 
                "./results/diffsel/wtDNA_ctrl/summary_FP16-02-500ug-meansitediffsel.csv", 
                "./results/diffsel/wtDNA_ctrl/summary_FP20-01-500ug-meansitediffsel.csv"
               ]
plotfile = './results/diffsel/Figures/Positive_diffsel_fig.pdf'
dms_tools2.plot.plotSiteDiffSel(names, diffselfiles, plotfile, "positive", maxcol=1, white_bg=True)
showPDF(plotfile)
```

```
/home/adingens/dms_tools2/dms_tools2/plot.py:746: FutureWarning: specifying 'categories' or 'ordered' in .astype() is deprecated; pass a CategoricalDtype instead
  categories=names)
```

In [3]:

```
names = ["VRC34.01", "vFP16.02", "vFP20.01"]
diffselfiles = ["./results/diffsel/wtDNA_ctrl/summary_VRC34-33ug-meansitediffsel.csv", 
                "./results/diffsel/wtDNA_ctrl/summary_FP16-02-500ug-meansitediffsel.csv", 
                "./results/diffsel/wtDNA_ctrl/summary_FP20-01-500ug-meansitediffsel.csv"
               ]
plotfile = './results/diffsel/Figures/Total_diffsel.pdf'
dms_tools2.plot.plotSiteDiffSel(names, diffselfiles, plotfile, "total", maxcol=1, white_bg=True)
showPDF(plotfile)
```

```
/home/adingens/dms_tools2/dms_tools2/plot.py:746: FutureWarning: specifying 'categories' or 'ordered' in .astype() is deprecated; pass a CategoricalDtype instead
  categories=names)
```

### Make logo plots visualizing the fraction surviving¶

The plots above summarize the average fraction surviving across the length of the gene with line plots. But the most comprehensive way to show this selection is in the form of logo plots that can be created with dms2\_logoplot.

In [31]:

```
groups = diffselbatch_wtDNActrl['group'].unique()
```

In [32]:

```
for antibody in groups:

    mutdiffsel = os.path.join(diffsel_wtDNActrl_dir, 'summary_{0}-meanmutdiffsel.csv'.format(antibody))
    # now create the logo plot with the overlay
    #scale bar unit is maximum effect
    mutdiffseldf = pd.read_csv(mutdiffsel)
    scaleunit = '10'
    #scaleunit = '{0:.1g}'.format(mutdiffseldf['mutdiffsel'].max())
    scalelabel = '"differential selection = {0}"'.format(scaleunit)
    logoplot = os.path.join(diffsel_wtDNActrl_dir, '{0}_diffsel.pdf'.format(antibody))
    print(logoplot)
    print("\nCreating logo plot for {0} from {1}".format(antibody, mutdiffsel))
    log = !dms2_logoplot \
            --diffsel {mutdiffsel} \
            --name {antibody} \
            --outdir {diffsel_wtDNActrl_dir} \
            --restrictdiffsel positive \
            --sepline no \
            --nperline 84 \
            --overlay1 {mutdiffsel} wildtype wildtype \
            --scalebar {scaleunit} {scalelabel} \
            --underlay yes \
            --use_existing {use_existing}
    #showPDF(logoplot)
```

```
./results/diffsel/wtDNA_ctrl/FP16-02-500ug_diffsel.pdf

Creating logo plot for FP16-02-500ug from ./results/diffsel/wtDNA_ctrl/summary_FP16-02-500ug-meanmutdiffsel.csv
./results/diffsel/wtDNA_ctrl/FP20-01-500ug_diffsel.pdf

Creating logo plot for FP20-01-500ug from ./results/diffsel/wtDNA_ctrl/summary_FP20-01-500ug-meanmutdiffsel.csv
./results/diffsel/wtDNA_ctrl/VRC34-33ug_diffsel.pdf

Creating logo plot for VRC34-33ug from ./results/diffsel/wtDNA_ctrl/summary_VRC34-33ug-meanmutdiffsel.csv
```

In [34]:

```
diffsel_wtvirusctrl_dir_totallogo = os.path.join(diffsel_wtDNActrl_dir, 'Total_Logoplots')
if not os.path.isdir(diffsel_wtvirusctrl_dir_totallogo):
    os.mkdir(diffsel_wtvirusctrl_dir_totallogo)

for antibody in groups:

    mutdiffsel = os.path.join(diffsel_wtDNActrl_dir, 'summary_{0}-meanmutdiffsel.csv'.format(antibody))
    # now create the logo plot with the overlay
    #scale bar unit is maximum effect
    mutdiffseldf = pd.read_csv(mutdiffsel)
    scaleunit = '{0:.1g}'.format(mutdiffseldf['mutdiffsel'].max())
    scalelabel = '"differential selection = {0}"'.format(scaleunit)
    logoplot = os.path.join(diffsel_wtDNActrl_dir, '{0}_All_diffsel.pdf'.format(antibody))
    #print(logoplot)
    print("\nCreating logo plot for {0} from {1}".format(antibody, mutdiffsel))
    log = !dms2_logoplot \
            --diffsel {mutdiffsel} \
            --name {antibody} \
            --outdir {diffsel_wtvirusctrl_dir_totallogo} \
            --restrictdiffsel all \
            --sepline yes \
            --nperline 84 \
            --overlay1 {mutdiffsel} wildtype wildtype \
            --scalebar {scaleunit} {scalelabel} \
            --underlay yes \
            --use_existing {use_existing}
    #showPDF(logoplot)
```

```
Creating logo plot for FP16-02-500ug from ./results/diffsel/wtDNA_ctrl/summary_FP16-02-500ug-meanmutdiffsel.csv

Creating logo plot for FP20-01-500ug from ./results/diffsel/wtDNA_ctrl/summary_FP20-01-500ug-meanmutdiffsel.csv

Creating logo plot for VRC34-33ug from ./results/diffsel/wtDNA_ctrl/summary_VRC34-33ug-meanmutdiffsel.csv
```

Now, we overlay the original mapping of VRC34.01 onto the logoplots Kong et al 2016. We took data from testing single BG505 (not T332N) point mutants in TZM-bl assays. This file contains the fold change in IC50 relative to wildtype for data pulled from tables S7 and S9 of Kong et al 2016, which we map ont the logoplots.

In [35]:

```
Kong_LogVRC34 = './data/BG505pseudovirus_VRC34.01_site_log2IC50foldchange.csv'
figuredir = "./results/diffsel/Figures/"

mutdiffsel = './results/diffsel/wtDNA_ctrl/summary_VRC34-33ug-meanmutdiffsel.csv'
mutdiffseldf = pd.read_csv(mutdiffsel)
scaleunit = '{0:.1g}'.format(mutdiffseldf['mutdiffsel'].max())
scalelabel = '"differential selection = {0}"'.format(scaleunit)
logoplot = './results/diffsel/Figures/VRC34-01-LogFoldChangeunderlay_diffsel.pdf'
antibody = "VRC34-01-LogFoldChangeunderlay"
VRC34name = " TZMbl"
print("\nCreating logo plot for {0} from {1}".format(antibody, mutdiffsel))
log = !dms2_logoplot \
        --diffsel {mutdiffsel} \
        --name {antibody} \
        --outdir {figuredir} \
        --restrictdiffsel all \
        --sepline yes \
        --nperline 84 \
        --overlaycolormap YlOrRd \
        --overlay1 {mutdiffsel} wildtype wildtype \
        --overlay2 {Kong_LogVRC34} {VRC34name} "Log fold change IC50" \
        --underlay yes \
        --scalebar {scaleunit} {scalelabel} \
        --use_existing {use_existing}

mutdiffsel = './results/diffsel/wtDNA_ctrl/summary_VRC34-33ug-meanmutdiffsel.csv'
mutdiffseldf = pd.read_csv(mutdiffsel)
scaleunit = "10"
#scaleunit = '{0:.1g}'.format(mutdiffseldf['mutdiffsel'].max())
scalelabel = '"differential selection = {0}"'.format(scaleunit)
logoplot = './results/diffsel/Figures/VRC34-01-LogFoldChangeunderlay-positive_diffsel.pdf'
antibody = "VRC34-01-LogFoldChangeunderlay-positive"
VRC34name = "TZMbl"
print("\nCreating logo plot for {0} from {1}".format(antibody, mutdiffsel))
log = !dms2_logoplot \
        --diffsel {mutdiffsel} \
        --name {antibody} \
        --outdir {figuredir} \
        --restrictdiffsel positive \
        --sepline no \
        --nperline 84 \
        --overlaycolormap YlOrRd \
        --overlay1 {mutdiffsel} wildtype wildtype \
        --overlay2 {Kong_LogVRC34} {VRC34name} "Log fold change IC50" \
        --underlay yes \
        --scalebar {scaleunit} {scalelabel} \
        --use_existing {use_existing}

showPDF(logoplot)
```

```
Creating logo plot for VRC34-01-LogFoldChangeunderlay from ./results/diffsel/wtDNA_ctrl/summary_VRC34-33ug-meanmutdiffsel.csv

Creating logo plot for VRC34-01-LogFoldChangeunderlay-positive from ./results/diffsel/wtDNA_ctrl/summary_VRC34-33ug-meanmutdiffsel.csv
```

## Make logoplots of key sites¶

Now, we will zoom in on the escape profile in regions of interest.

In [36]:

```
keysites = list(range(85, 91)) + list(range(227, 244)) + list(range(512, 525))

# Env sites numbers are strings because some have trailing letters (e.g., 100A)
keysites = list(map(str, keysites))
```

In [37]:

```
#import rpy2.robjects
import natsort
#diffsel_wtDNActrl_dir
names = ["VRC34.01", "vFP16.02", "vFP20.01"]
diffselfiles = ["./results/diffsel/wtDNA_ctrl/summary_VRC34-33ug-meanmutdiffsel.csv", 
                "./results/diffsel/wtDNA_ctrl/summary_FP16-02-500ug-meanmutdiffsel.csv", 
                "./results/diffsel/wtDNA_ctrl/summary_FP20-01-500ug-meanmutdiffsel.csv"
               ]

mutdiffsels = [] #list of csv files
for mutdiffsel, name in zip(diffselfiles, names): 
    pandadf = pd.read_csv(mutdiffsel)
    # read data frame from tidy CSV format to wide form
    df = dms_tools2.diffsel.tidyToWide(pandadf, valuecol='mutdiffsel')
    
    # only show positive values of mutdiffsel
    df[dms_tools2.AAS] = df[dms_tools2.AAS].clip(lower=0)
    
    #sort sites by 
    df = df.reindex(index=natsort.order_by_index(df.index,natsort.index_natsorted(df.site)))
    
    # add a column indicating what we should show
    df['show'] = df['site'].isin(keysites)
    
    # want to indicate wildtype along with site number, e.g, A512
    df['site'] = df['wildtype'] + df['site']
    # name of file to plot
    zoomlogoplot = os.path.join(diffsel_wtDNActrl_dir, '{0}_zoomed_diffsel.pdf'.format(name))
    # plot and show
    dms_tools2.rplot.siteSubsetGGSeqLogo(
            logodata=df,
            chars=dms_tools2.AAS,
            plotfile=zoomlogoplot,
            width=8,
            height=2.5,
            yname='differential selection',
            ylimits=(0,31),
            )
    showPDF(zoomlogoplot)
```

Now, we are zooming in on sites under negative selection across each antibody.

In [38]:

```
diffsel_wtDNActrl_dir = os.path.join(diffseldir, 'wtDNA_ctrl')

## Looking at negative selection
keysites = list(range(88, 91)) + list(range(611, 614)) + list(range(621, 626))

# Env sites numbers are strings because some have trailing letters (e.g., 100A)
keysites = list(map(str, keysites))

#import rpy2.robjects
import natsort
#diffsel_wtDNActrl_dir
names = ["VRC34.01", "vFP16.02", "vFP20.01"]
diffselfiles = ["./results/diffsel/wtDNA_ctrl/summary_VRC34-33ug-meanmutdiffsel.csv", 
                "./results/diffsel/wtDNA_ctrl/summary_FP16-02-500ug-meanmutdiffsel.csv", 
                "./results/diffsel/wtDNA_ctrl/summary_FP20-01-500ug-meanmutdiffsel.csv"
               ]

mutdiffsels = [] #list of csv files
for mutdiffsel, name in zip(diffselfiles, names): 
    pandadf = pd.read_csv(mutdiffsel)
    #print(pandadf)
    # read data frame from tidy CSV format to wide form
    df = dms_tools2.diffsel.tidyToWide(pandadf, valuecol='mutdiffsel')
    
    # only show negative values of mutdiffsel
    df[dms_tools2.AAS] = df[dms_tools2.AAS].clip(upper=0)
    
    #sort sites by 
    df = df.reindex(index=natsort.order_by_index(df.index,natsort.index_natsorted(df.site)))
    
    # add a column indicating what we should show
    df['show'] = df['site'].isin(keysites)
    
    
    # want to indicate wildtype along with site number, e.g, A512
    df['site'] = df['wildtype'] + df['site']
    # name of file to plot
    zoomlogoplot = os.path.join(diffsel_wtDNActrl_dir, '{0}_zoomed_negative_diffsel.pdf'.format(name))
    #print(df)
    # plot and show
    dms_tools2.rplot.siteSubsetGGSeqLogo(
            logodata=df,
            chars=dms_tools2.AAS,
            plotfile=zoomlogoplot,
            width=3.5,
            height=2.5,
            yname='negative differential selection',
            ylimits=(-33,0),
            )
    showPDF(zoomlogoplot)
```
